# Chiral Linker Installation in a Metal–Organic Framework for Enantioselective Luminescent Sensing

**DOI:** 10.1021/jacs.4c03728

**Published:** 2024-05-22

**Authors:** Zongsu Han, Tiankai Sun, Rong-Ran Liang, Yifan Guo, Yihao Yang, Mengmeng Wang, Yue Mao, Peter R. Taylor, Wei Shi, Kun-Yu Wang, Hong-Cai Zhou

**Affiliations:** † Department of Chemistry, Texas A&M University, College Station, Texas 77843, United States; ‡ Frontiers Science Center for New Organic Matter, Key Laboratory of Advanced Energy Materials Chemistry (MOE), and State Key Laboratory of Advanced Chemical Power Sources, College of Chemistry, Nankai University, Tianjin 300071, China; § School of Pharmaceutical Science and Technology, Tianjin University, Tianjin 300072, China

## Abstract

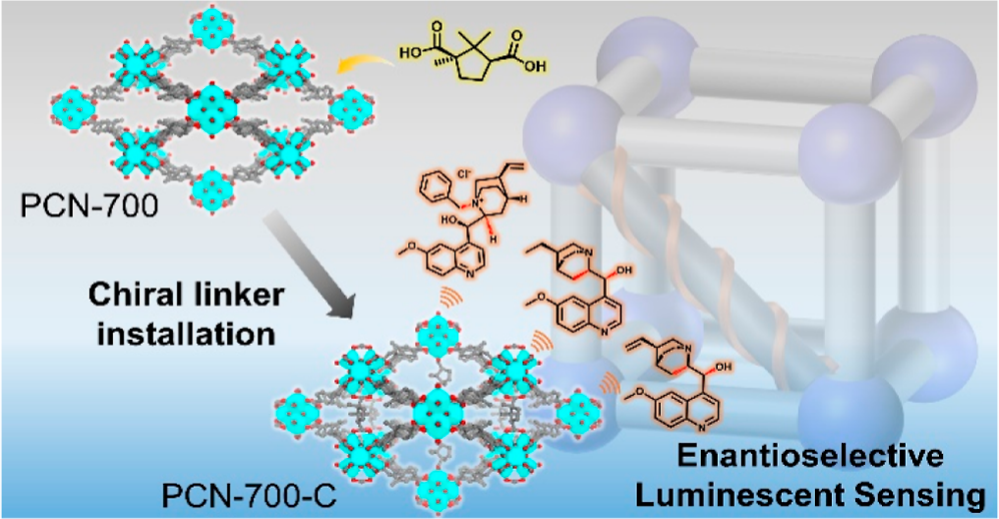

Linker installation is a potent strategy for integrating specific properties and functionalities into metal–organic frameworks (MOFs). This method enhances the structural diversity of frameworks and enables the precise construction of robust structures, complementing the conventional postsynthetic modification approaches, by fully leveraging open metal sites and active organic linkers at targeting locations. Herein, we demonstrated an insertion of a d-camphorate linker into a flexible Zr-based MOF, PCN-700, through linker installation. The resultant homochiral MOF not only exhibits remarkable stability but also functions as a highly efficient luminescent material for enantioselective sensing. Competitive absorption and energy/electron transfer processes contribute to the sensing performance, while the difference in binding affinities dominates the enantioselectivity. This work presents a straightforward route to crafting stable homochiral MOFs for enantioselective sensing.

## Introduction

Advancements in modern science and pharmaceutics have intensified the focus on medication purity, particularly regarding the optical purity toward chiral drugs.^1,2^ Enantiomers, as distinct mirrored forms of a molecule, often exhibit varying effects on the human body,^3,4^ especially for their different therapeutical performance and toxic side effects.^5,6^ Despite significant progress in analytical instrumentation, the real-time differentiation of enantiomers remains a grand challenge, attributed to their highly similar properties.^7,8^ Nevertheless, such differentiation is of upmost importance in regulating the manufacturing and usage of pharmaceuticals and pesticides. Recently, luminescent sensing has been recognized as a promising approach for distinguishing enantiomers, noted for its selectivity, sensitivity, visual cues, and rapid response.^9−11^ However, integrating chiral centers into luminescent materials through conventional synthetic methods encounters challenges, primarily due to the inherent symmetry constraints in solid-state materials.

Metal–organic frameworks (MOFs) are nanoporous materials with unique merits in structural diversity and readily modifiable backbones.^12−14^ Such versatility allows for targeted synthesis or modification of MOFs to meet the practical requirements of sensing applications.^15,16^ The effectiveness of MOF-based luminescent materials is evidenced by their successful applications in detecting a wide range of substances, including ions, organic solvents, volatile organic compounds, persistent organic pollutants, and micromolecular biomarkers.^17−20^

**Scheme 1:**
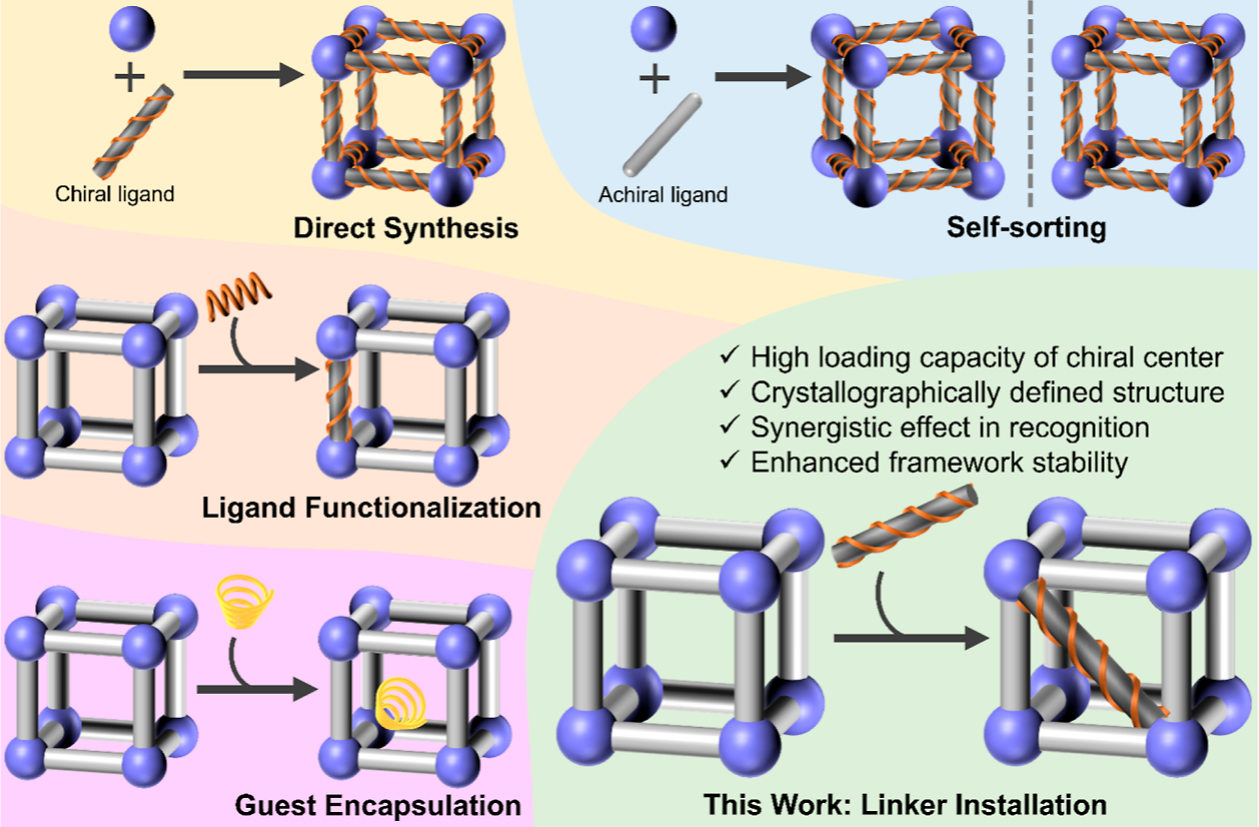
Construction Methods of MOF-Based Enantioselective Sensing Materials

To construct MOF-based enantioselective sensing materials, in general, chiral centers can be introduced through direct synthesis, self-sorting, ligand functionalization, and guest encapsulation (Scheme 1). In the direct synthesis and self-sorting approaches, it is challenging to design and predict the crystallization processes,^21−25^ which often requires extensive experimentation. Regarding enantioselective sensors constructed through ligand functionalization,^26,27^ their application potentials are limited by the MOFs’ pore size and stability, which in turn restricts the range of feasible reactions and the quantity of incorporated molecules. For guest encapsulation,^28−30^ the introduced chiral centers would be displaced by ions/molecules with the same charge, which may cause the reduction of certain performance.

In contrast to the aforementioned approaches, linker installation has demonstrated its capability to intricately position diverse properties and functionalities within MOFs under mild conditions, evidenced in a range of MOFs containing coordinatively unsaturated Zr-clusters, like PCN-700 and PCN-608.^31−34^ Through careful matching of missing-linker cavities and bridging ligands, the installed linkers can reach high occupancies at the potential binding sites, enabling the unassailable characterization of the final structure by single-crystal X-ray diffraction. On the contrary, traditional postsynthetic modification is inefficient, leading to highly disordered or amorphous structures. Moreover, the inserted linker is highly inert, ensured through its dual-end linkage. In this work, we developed a new MOF-based enantioselective sensing material, PCN-700-C, constructed by the linker installation using a commercially available chiral molecule, d-camphoric acid, on PCN-700. PCN-700-C exhibits remarkable stability and enantioselectivity in the luminescent sensing of a variety of chiral drugs (Figure S1). Additionally, the enantiomeric excess (ee) values of the chiral molecule mixtures can be easily ascertained from changes in luminescence intensity, while the introduced d-camphorate remains intact within the framework after the sensing process. Competitive absorption (CA) of the excitation energy and energy/electron transfer processes contribute to the sensing performance, while the different binding abilities are dedicated to the enantioselectivity of this chiral MOF.

## Results and Discussion

### Structures and Characterizations

**Figure 1:**
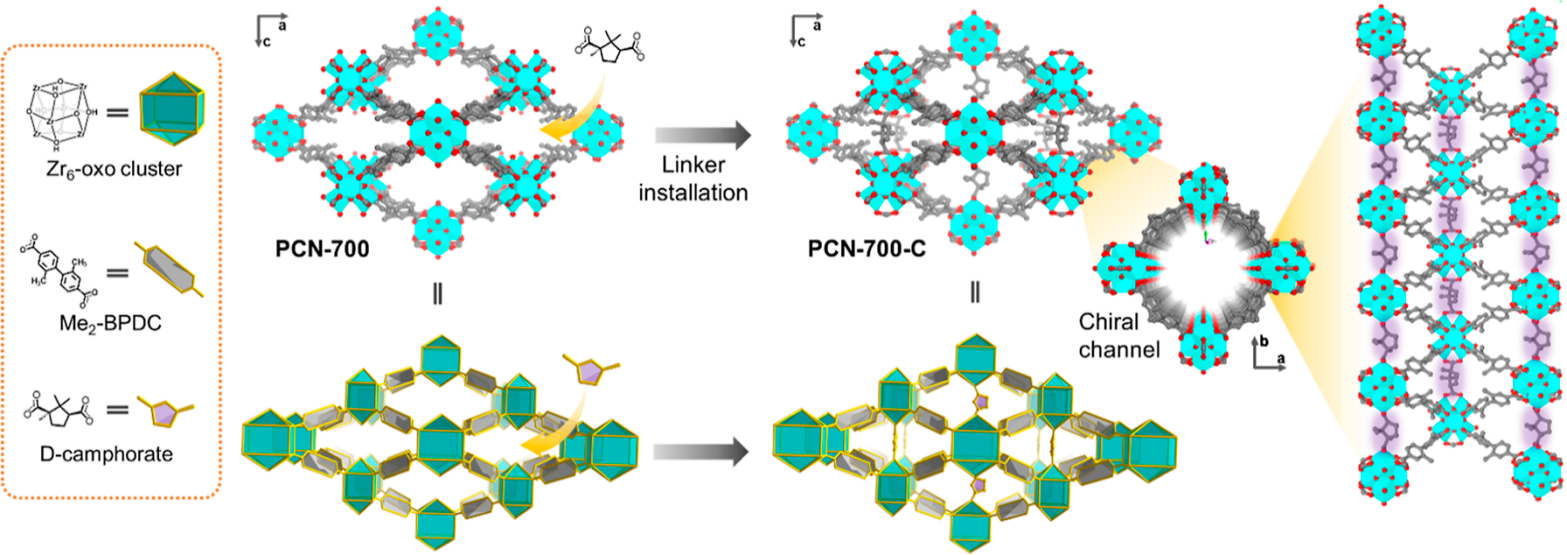
Construction process of PCN-700-C through chiral linker installation.

PCN-700 is a highly flexible MOF bearing a coordinatively unsaturated Zr_6_ cluster.^31,32^ Compared with the classical MOF UiO-67 featuring 12-connected Zr_6_ clusters that reach coordinative saturation,^35^ PCN-700 possessing 8-connected Zr_6_ clusters allows for the successful installation of a variety of ligands with different lengths within its two distinct missing-linker pockets.^31,32^d-Camphoric acid is commercially available at a very low cost, which has been widely used for over a century.^36^ Furthermore, the coordination capability of d-camphoric acid is similar to the ligand of PCN-700, according to the hard–soft-acid–base theory, while their molecular lengths and configurations are varied, hindering the occurrence of the ligand exchange process. In this study, d-camphorate was chosen as the linker for installation because it features two carboxyl groups at an appropriate distance fitting one of the missing-linker pockets in PCN-700. The successful incorporation of d-camphorate was confirmed by single-crystal X-ray diffraction (Figure 1). As a result, the two carboxyl groups of d-camphorate were fixed within two adjacent Zr-clusters and caused the shrink of the *c* axis from 14.7 to13.9 Å (Table S1).

Powder X-ray diffraction (PXRD) patterns showed that the framework structure was intact during the linker installation procedure (Figure S2). ^1^H nuclear magnetic resonance (NMR) spectroscopy of the digested sample confirmed the existence of d-camphoric acid (Figure S3). Thermogravimetric analysis of PCN-700-C showed a continuous mass loss process due to the presence of solvents in its pores (Figure S4). pH and solvent stabilities of PCN-700-C were confirmed by soaking the samples in various solvents for 10 h, while PCN-700-C remained stable in most common solvents and aqueous solutions with pH ranging from 2 to 12 (Figures S5 and S6) and can preserve partial crystallinity in pH 0–1 (Figure S5). The time-dependent luminescence intensities of PCN-700-C in DMF were stable (Figure S7), demonstrating its suitability for practical sensing.

### Luminescence Sensing

The enantioselective sensing performance of PCN-700-C was studied through three pairs of chiral drug epimers and phase transfer catalysts,^37−39^*N*-benzylquininium chloride (1*R*) and *N*-benzylquinidinium chloride (1*S*), hydroquinine (2*R*) and hydroquinidine (2*S*), quinine (3*R*) and quinidine (3*S*) (Figure S1). The luminescence intensities of PCN-700-C at 330 nm excited at 290 nm distinctly decreased with the gradual addition of the analytes, while the luminescence intensities of the analytes at ∼350–360 nm increased (Figures S8–S10). The emission intensities of the ligand at 330 nm show different quenching efficiencies toward the epimers (Figures S11–S13). The luminescence quenching efficiency can be quantitatively described by the exponential nonlinear S–V equation *I*_0_/*I* = *a* exp(*k*[*C*]) + *b*,^40,41^ where *a*, *b*, and *k* are constants. At the same time, the emission intensities of the epimers at ∼350–360 nm exhibited similar intensity changes, which excluded the interference from analyte emission toward the selectivity.

**Figure 2:**
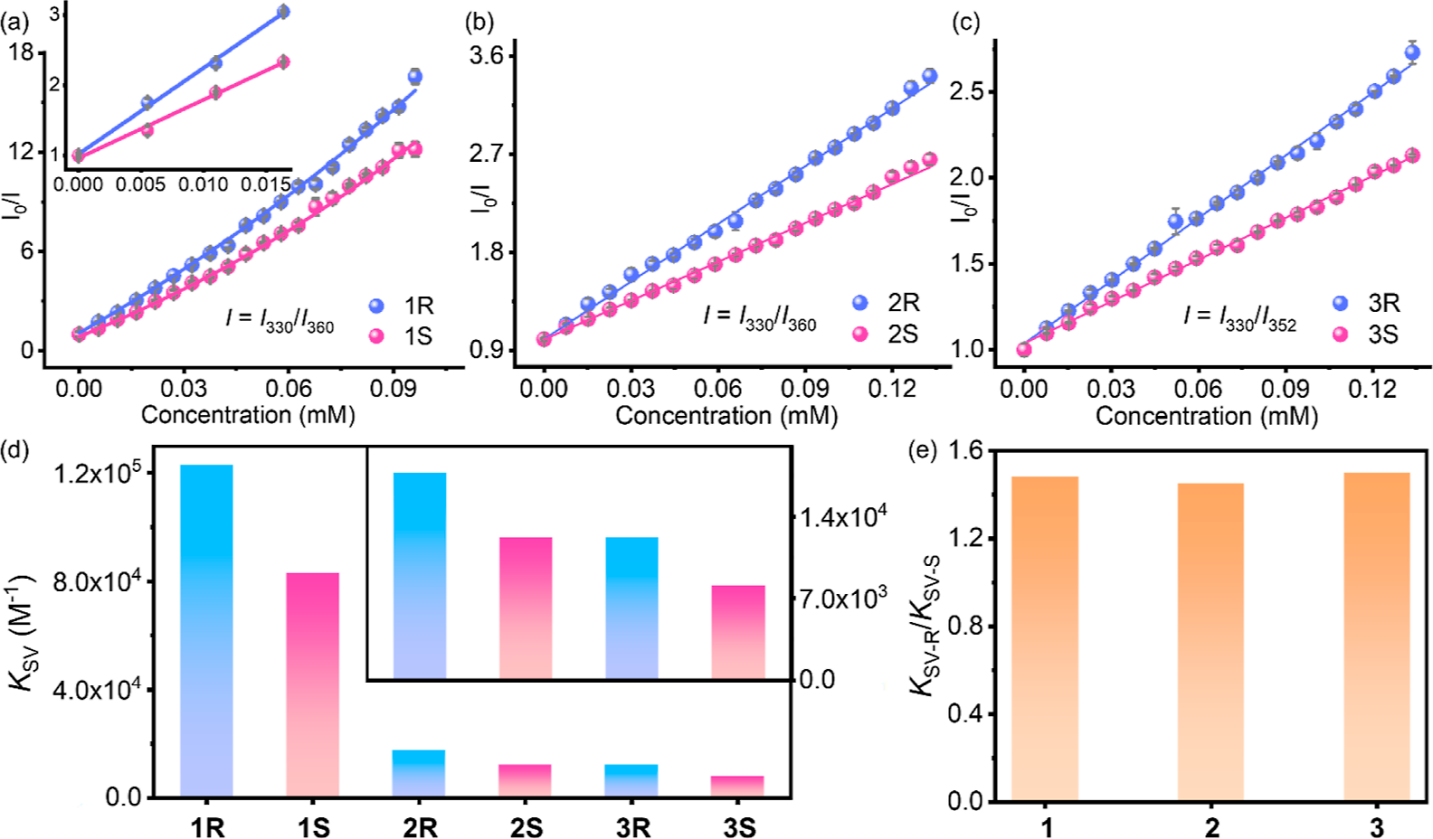
Quenching efficiencies of PCN-700-C toward 1*R*/1*S* (a), 2*R*/2*S* (b), 3*R*/3*S* (c), the calculated *K*_SV_ values (d), and the selectivities (e).

Furthermore, given the close peaks of the ligand and analytes, we found that the ratio of their emission intensity can better describe the intensity changes, with a higher correlation coefficient (Figure 2a–c). For 1*R*/1*S*, the ratiometric changes of *I*_330_/*I*_360_ can be described by the nonlinear S–V equation; while for 2*R*/2*S* and 3*R*/3*S*, the ratiometric changes of *I*_330_/*I*_360_ and *I*_330_/*I*_352_ can be described by the linear S–V equation *I*_0_/*I* = *k*[*C*] + *b*,^40,41^ where *k* and *b* are constants. The *K*_SV_ values of 1*R*/2*R*/3*R* are 1.48/1.45/1.50 times larger than their epimers, respectively (Figure 2d,e). All the fitting results are provided in the Supporting Information. Besides, all the emission signals become stable within 10 s (Figures S14–S19), confirming PCN-700-C’s capability for rapid luminescence sensing.

**Figure 3:**
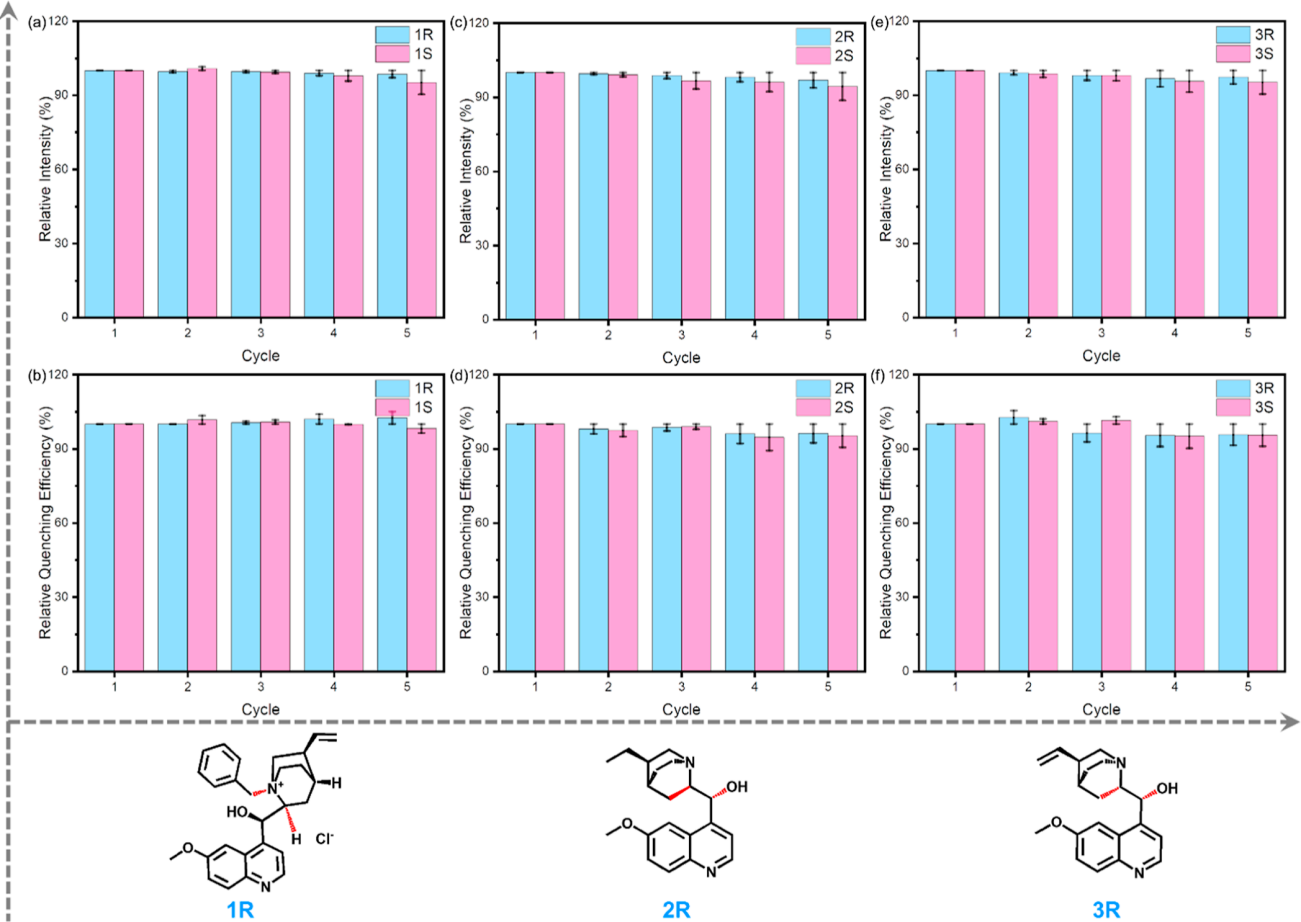
Intensities and quenching efficiencies of the recycling experiments for PCN-700-C toward 1*R*/1*S* (a,b), 2*R*/2*S* (c,d), and 3*R*/3*S* (e,f).

Recycling experiments were also tested (Figures S20–S22), demonstrating that both the intensities and the quenching efficiencies can remain stable through five cycles, with less than 5% changes (Figure 3). ^1^H NMR spectra of the digested PCN-700-C after sensing experiments demonstrate that there is almost no change in the ratio between d-camphorate and the MOF ligand (Figure S23), further confirming the stability of this sensing material.

**Figure 4:**
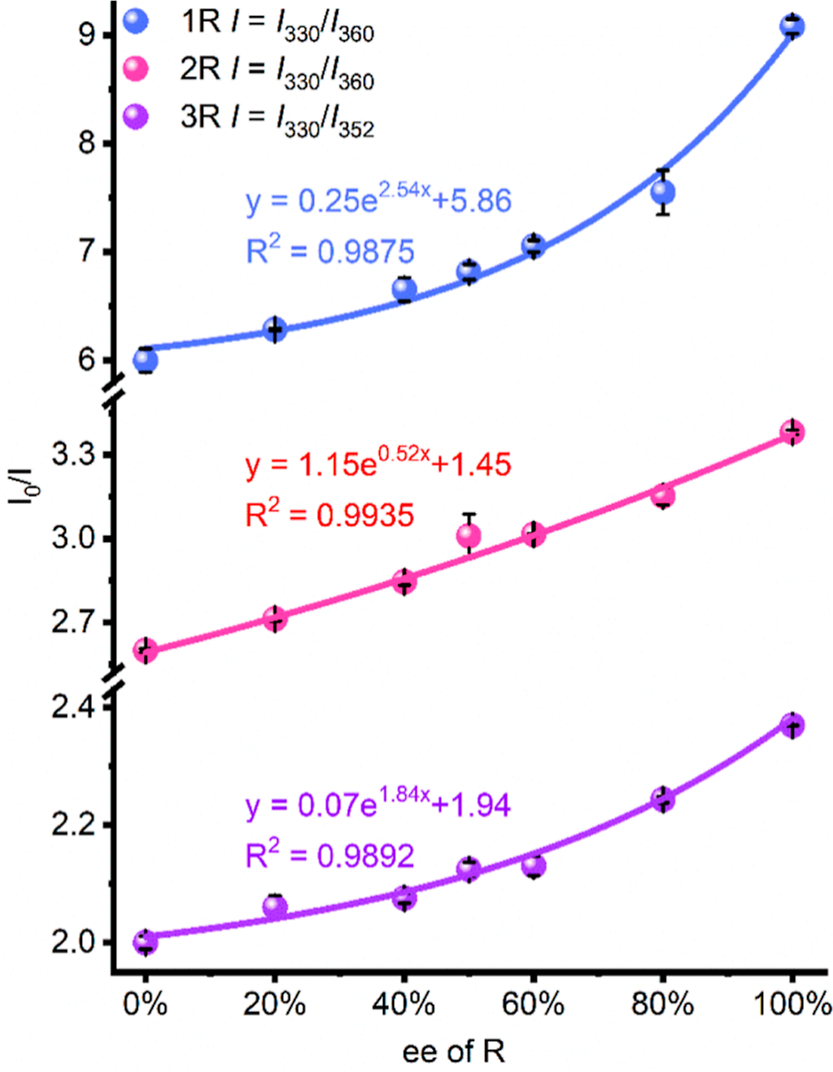
Intensity changes of PCN-700-C toward different ee values of the analyte mixtures.

For sensing the epimer mixture, the ee values can also be obtained based on the different quenching behaviors of PCN-700-C. The ee value is defined as (*a* – *b*)/(*a* + *b*) × 100%, where *a* and *b* are the concentrations of different enantiomers. The total concentration (*a* + *b*) can be calculated by *m*/*MV*, where *m* is the mass of the analyte, *V* is the volume, and *M* is the molecular mass of the analyte. The emission intensities of PCN-700-C upon the addition of mixtures with different ee values were measured (Figures S24–S26). The changes in the intensities can be well fitted by exponential-type equations with different ee values (Figure 4), confirming that PCN-700-C is an effective sensor to quantitatively determine the components of the mixture.

### Sensing Mechanism

**Figure 5:**
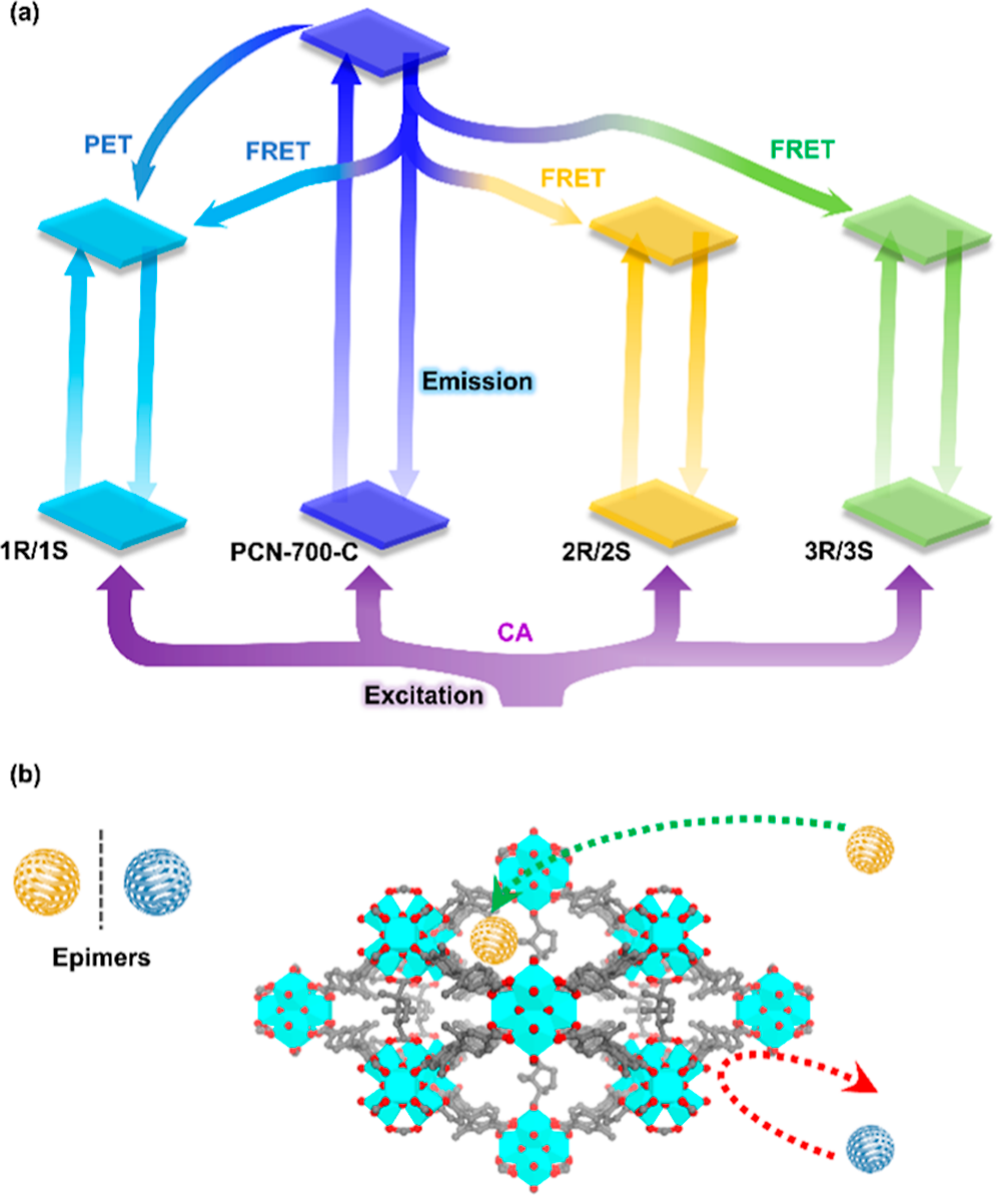
Sensing mechanism (a) and selective sensing mechanism (b) of PCN-700-C toward the analytes.

The sensing mechanism was studied through a series of characterizations. Based on the ultraviolet–visible (UV–vis) absorption spectrum of the ligand and the phosphorescence spectrum of PCN-700-C at 77 K (Figures S27 and S28), the calculated singlet-state and triplet-state energy gaps of the ligand are higher than 5000 cm^–1^ (Figure S29), inducing an intersystem crossing process.^40^ PXRD patterns and infrared spectra reveal no obvious structural collapse of PCN-700-C after the sensing experiments (Figures S30 and S31), indicating that the sensing function can be attributed to the property of PCN-700-C. There are obvious overlaps between the UV–vis absorption of the analytes and PCN-700-C, indicating the CA of the excitation light (Figure S32).^42^ There are also overlaps between the UV–vis absorption of the analytes and the emission spectrum of PCN-700-C, indicating the absence of the Förster resonance energy transfer process (Figure S32), which occurs when the ligand returns from the excited state to the ground state, and simultaneously, the analyte is promoted to the excited state through the nonradiative energy transfer.^42^ The lowest unoccupied molecular orbital (LUMO) energy levels of the ligand and the analytes were calculated, which shows that the LUMO energy level of 1*R* is lower than the energy level of 2*R*/3*R* and the ligand (Figure S33), demonstrating that the photoinduced electron transfer mechanism is present in the detection of 1*R*, which occurs when a photoelectron is transferred from the excited ligand to the ground-state analyte, but absent in the recognition of 2*R*/3*R* (Figure 5a).^42^ This extra mechanism may be the reason for the lower *K*_SV_ values of 2*R*/3*R* compared with 1*R* (∼1/7 and 1/10).

The lifetime changes of PCN-700-C with the additions of the analytes were measured (Figures S34–S36), and no obvious variations were observed (Figure S37), indicating a static quenching process, caused by the binding between the MOF and analytes.^40,41^

As for the selective sensing (Figure 5b), density functional theory calculations were carried out, which show that the bindings of PCN-700-C with R-epimers were more favored in energy than those with *S*-epimers. The combination energy differences between the d-camphorate linker and 1*R*/1*S*, 2*R*/2*S*, and 3*R*/3*S* are 5.74, 6.65, and 8.80 kcal/mol, respectively (Figure S38), indicating the stronger combination with *R*-epimers. Additionally, ^1^H NMR spectra of identical quantities of PCN-700-C, after immersion under the same concentrations of the analytes, were also measured for comparison (Figure S39). Notably, PCN-700-C adsorbed more *R*-epimers than *S*-epimers over the same duration. Such differential adsorption, which can be attributed to the different binding abilities for the epimers toward the d-camphorate linker, may explain the higher quenching efficiencies of PCN-700-C toward *R*-epimers.

## Conclusions

In summary, we have successfully developed a chiral sensing material, PCN-700-C, employing a linker installation strategy centered around Zr-clusters with high pH and solvent stabilities. The sophisticatedly engineered PCN-700-C material exhibits outstanding enantioselective sensing capabilities, along with precise quantification of both enantiomers and their mixtures (Table S2). CA of the excitation light and energy/electron transfer processes cause the quenching of PCN-700-C toward the epimers, while the different binding abilities contribute to the enantioselectivity. This study presents a convenient and cost-effective approach to constructing a robust chiral MOF-based luminescent sensing materials, capitalizing on the synergistic effects between the MOF’s tailored pocket and the chiral coordinative molecule.

## Supporting Information Available

Materials and methods, basic characterizations, luminescence sensing, sensing mechanism, and relative tables (PDF)
