# Supplementary material for: Chiral Linker Installation in a Metal–Organic Framework for Enantioselective Luminescent Sensing
Source: J Am Chem Soc. 2024 May 22;146(22):15446–52. doi: 10.1021/jacs.4c03728 (PMC11157530; doi:10.1021/jacs.4c03728)
Supplement: Supplementary file 1 — ja4c03728_si_001.pdf [file ja4c03728_si_001.pdf]

Supporting Information

Chiral Linker Installation in a Metal-Organic Framework for Enantioselective Luminescent Sensing

Zongsu Han,[†,§] Tiankai Sun,[‡,§] Rong-Ran Liang,[†,§] Yifan Guo,[⊥] Yihao Yang,[†] Mengmeng Wang,[‡] Yue Mao,[‡] Peter R. Taylor,[⊥] Wei Shi,[‡,*] Kun-Yu Wang,[†,*] and Hong-Cai Zhou[†,*]

[†]Department of Chemistry, Texas A&M University, College Station, Texas 77843, United States

[‡]Frontiers Science Center for New Organic Matter, Key Laboratory of Advanced Energy Materials Chemistry (MOE), and State Key Laboratory of Advanced Chemical Power Sources, College of Chemistry, Nankai University, Tianjin 300071, China

[⊥]School of Pharmaceutical Science and Technology, Tianjin University, Tianjin 300072, China

Contents

Materials and Methods	2
Basic Characterizations	4
Luminescence Sensing	8
Sensing Mechanism	18
Tables	26
References	29

Materials and Methods

Materials.

All the reagents were commercially available and used without further purification. Liquid ^{1}H NMR spectra were recorded on a Bruker AV 400 MHz and a Bruker Avance NEO 400 NMR spectrometer. For MOFs, about 3 mg samples were dissolved in 10 μL DCl and 0.5 mL d_6-DMSO. SCXRD patterns were collected by a Bruker-Axs Venture Ius Cmos Kappa X-ray Apex2 diffractometer with Cu-Kα radiation. The structures were solved by SHELXS (direct methods) and refined by SHELXL (full matrix least-squares techniques) in the Olex2 package.[1,2] PXRD measurements were performed using a Rigaku SmartLab SE X-ray diffractometer and a Bruker Powder-ECO X-ray diffractometer with Cu-Kα radiation. TGA data were obtained under nitrogen atmosphere on a Mettler Toledo TGA/DSC 1 thermogravimetric analyzer from 40 °C to 800 °C. Luminescence spectra, luminescence lifetimes, and phosphorescence spectrum at 77 K were recorded on an Edinburgh FS5 fluorescence spectrophotometer. UV-vis absorption spectra were measured by a Shimadzu UV-2450 spectrometer. IR spectra were tested by a Bruker Alpha II spectrometer.

Synthesis.

PCN-700 was synthesized referring to the literature.[3] 10 mg PCN-700 was soaked in 10 mL DMF solution of 20 mg D-camphoric acid, and heated at 80 ℃ for three days. After cooling down to room temperature, the samples were washed with fresh DMF for six times and soaked in fresh DMF for a night to obtain PCN-700-C. From the ^{1}H NMR spectra of the digested PCN-700-C, the ratio of D-camphoric acid and the ligand is ~3:1, which is little more than the 4:1 from the SCXRD data, even after long time soaking in fresh DMF. The extra D-camphoric acid should be coordinated to other open metal sites on the Zr-cluster with only one end but not installed with two ends, which is hard to be solved through SCXRD.

Luminescence sensing experiments.

PCN-700-C was grinded into fine powder before use. The samples for luminescence sensing and lifetimes experiments were dispersed in DMF by ultrasound for 10 minutes to form a suspension with a concentration of 0.3 mg mL^{-1}. For the recycling tests, MOFs were centrifuged and washed with fresh DMF for three times. For the phosphorescence

spectrum at 77 K, ~20 mg solid samples were added into an NMR tube and placed in the low temperature module of the equipment.

Fitting results in Figure 2.

(a)	1R	$y = 15.37e^{7.07x}-14.22$	$R^2 = 0.9976$;
	1S	$y = 16.18e^{5.67x}-15.34$	$R^2 = 0.9979$;
Inset:	1R	$y = 122.79x+1.02$	$R^2 = 0.9955$;
	1S	$y = 83.09x+0.96$	$R^2 = 0.9915$;
(b)	2R	$y = 17.70x+1.00$	$R^2 = 0.9968$;
	2S	$y = 12.21x+0.98$	$R^2 = 0.9965$;
(c)	3R	$y = 12.16x+1.03$	$R^2 = 0.9965$;
	3S	$y = 8.11x+1.04$	$R^2 = 0.9974$.

Fitting results in Figure S11.

1R	$y = 2.36e^{4.82x}-1.20$	$R^2 = 0.9770$;
1S	$y = 0.78e^{8.09x}+0.13$	$R^2 = 0.9790$.

Fitting results in Figure S12.

2R	$y = 1.25e^{7.37x}-0.25$	$R^2 = 0.9973$;
2S	$y = 0.74e^{9.04x}+0.24$	$R^2 = 0.9978$.

Fitting results in Figure S13.

3R	$y = 8.07e^{1.51x}-7.01$	$R^2 = 0.9875$;
3S	$y = 1.26e^{5.40x}-0.20$	$R^2 = 0.9914$.

Basic Characterizations

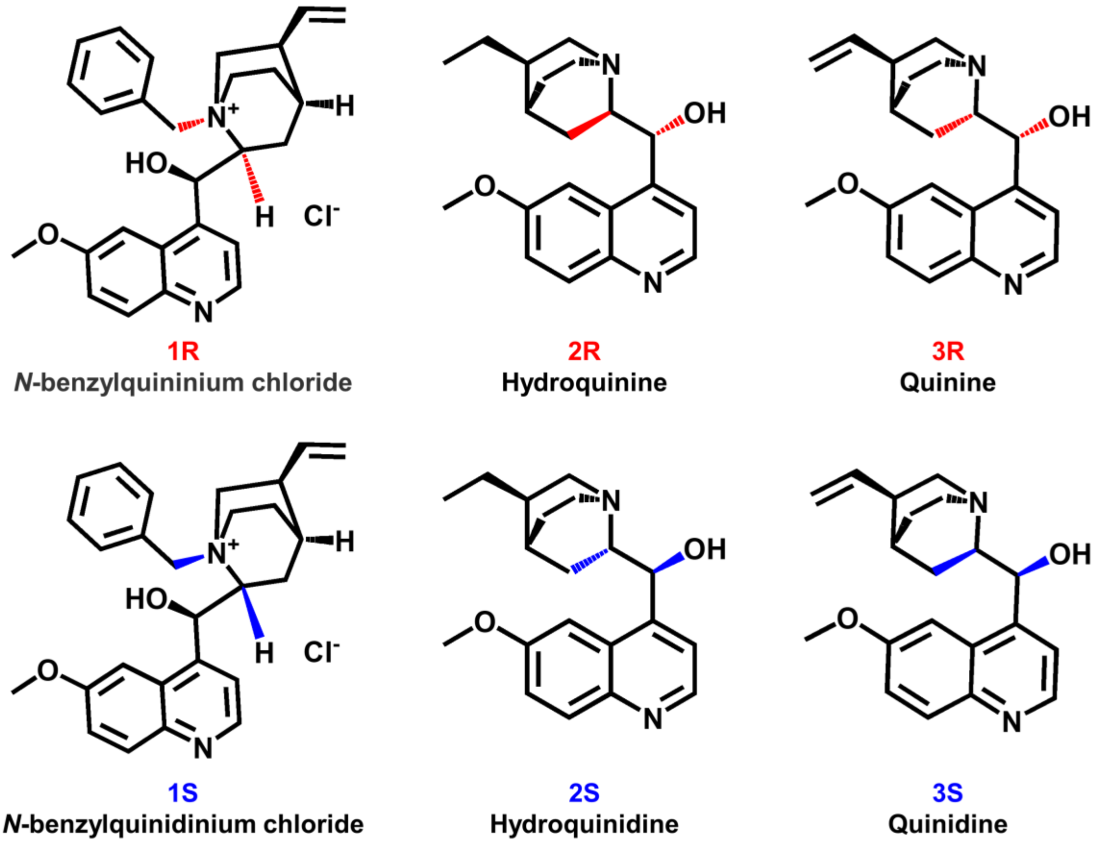

Figure S1. Structures of the selected epimers.

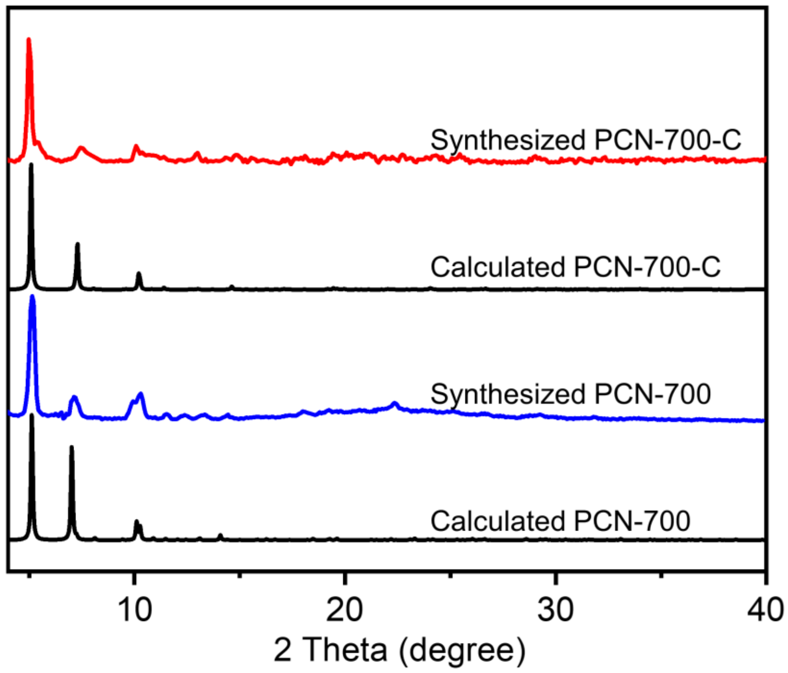

Figure S2. PXRD patterns of the calculated and synthesized PCN-700 and PCN-700-C.

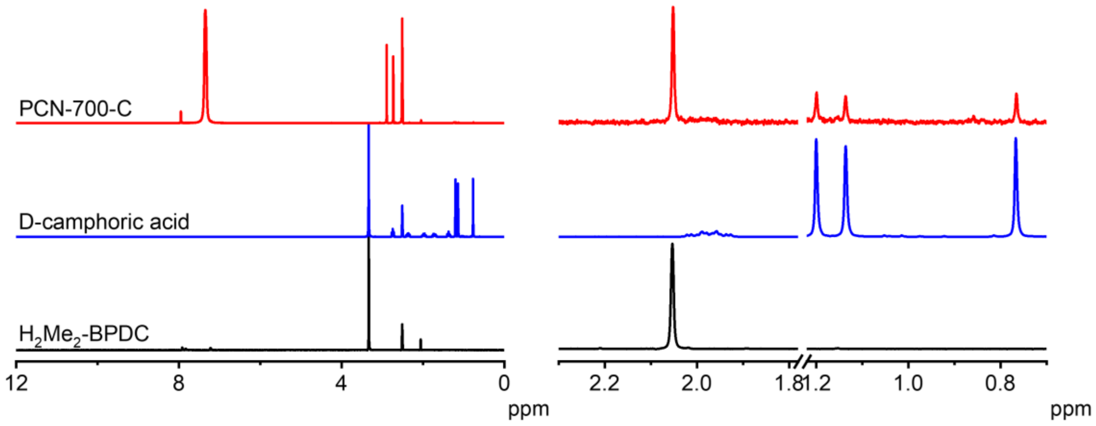

Figure S3. ^{1}H NMR spectra of the ligand H_2Me_2-BPDC, D-camphoric acid, and PCN-700-C.

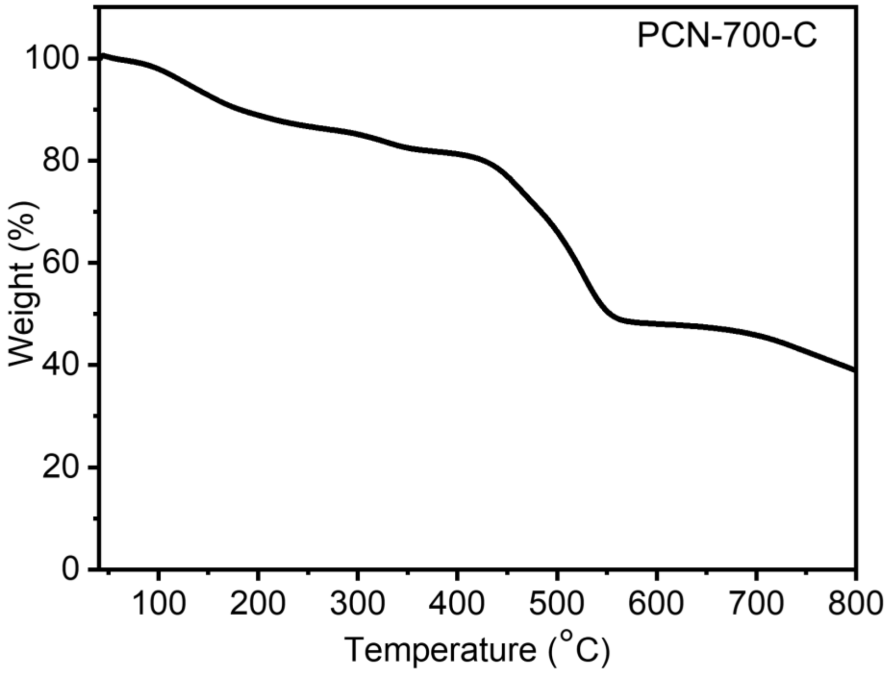

Figure S4. TGA curve of PCN-700-C.

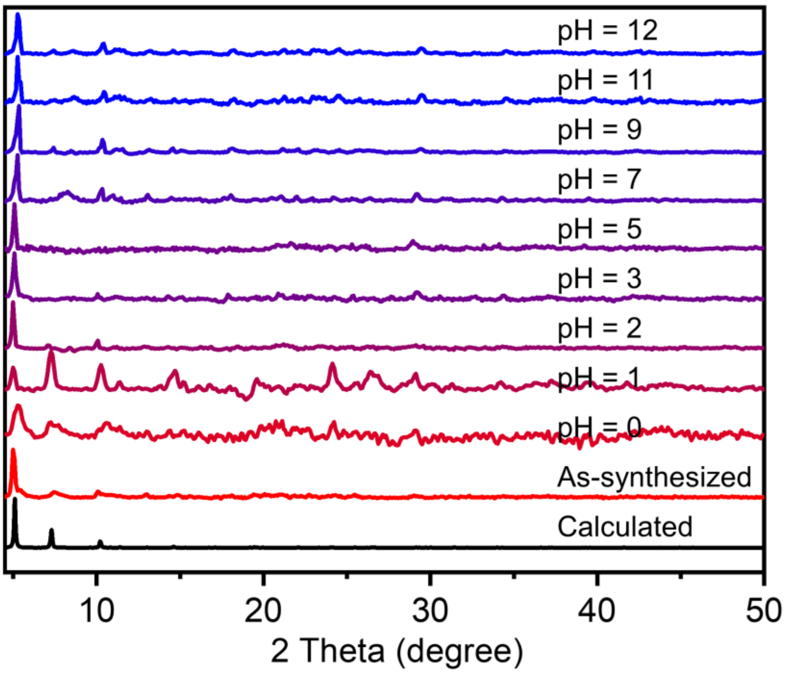

Figure S5. PXRD patterns of PCN-700-C soaking in different pH hydrochloric acid / sodium hydroxide aqueous solution for 10 hours.

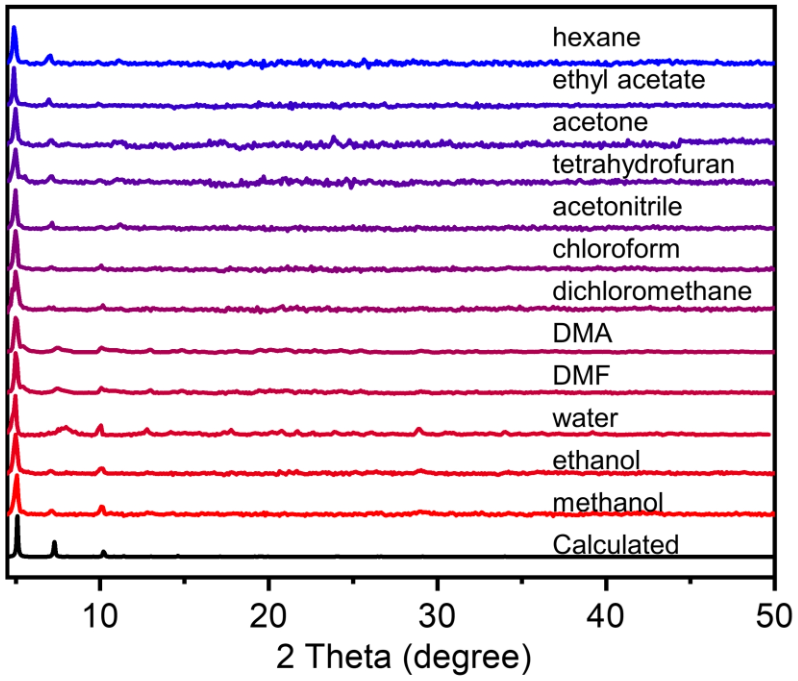

Figure S6. PXRD patterns of PCN-700-C soaking in different solvents for 10 hours.

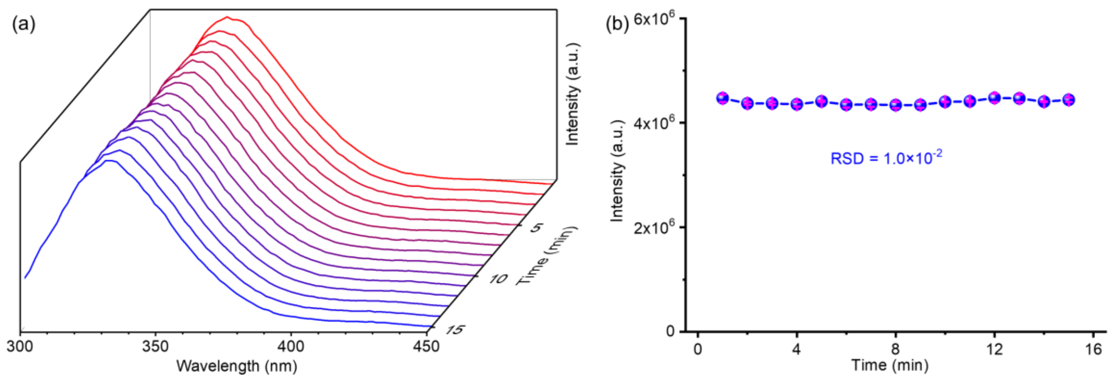

Figure S7. Time-dependent luminescence spectra (a) and intensities (b) of PCN-700-C in DMF.

Luminescence Sensing

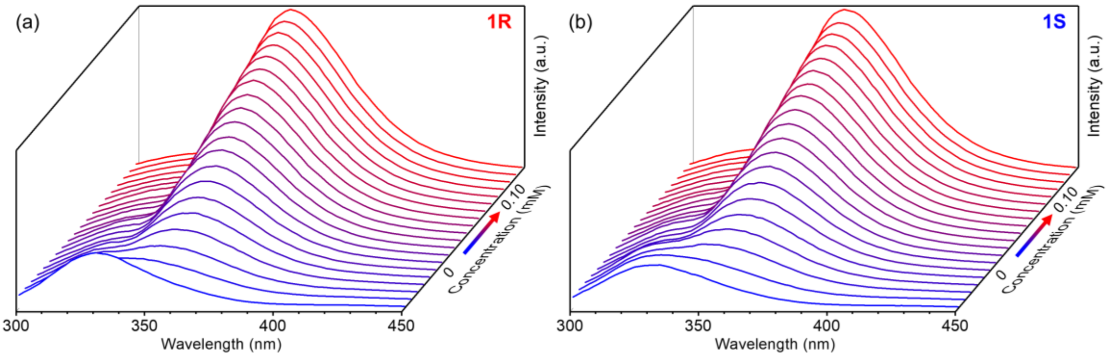

Figure S8. Luminescence spectra of PCN-700-C with the additions of 1R (a) and 1S (b).

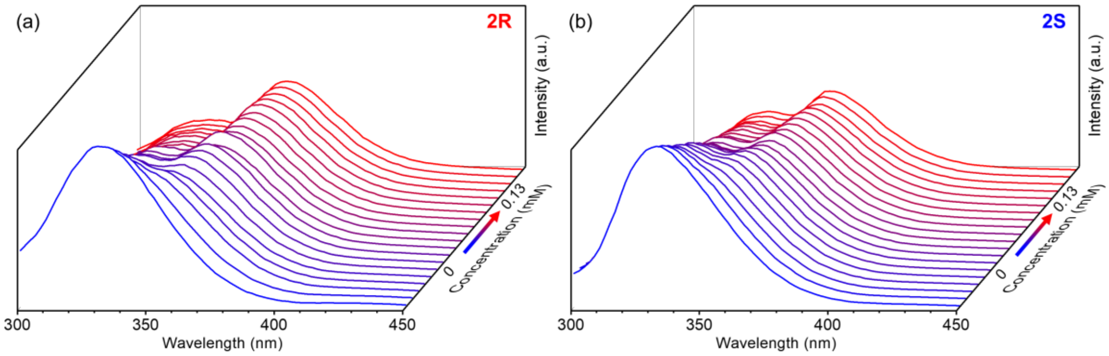

Figure S9. Luminescence spectra of PCN-700-C with the additions of 2R (a) and 2S (b).

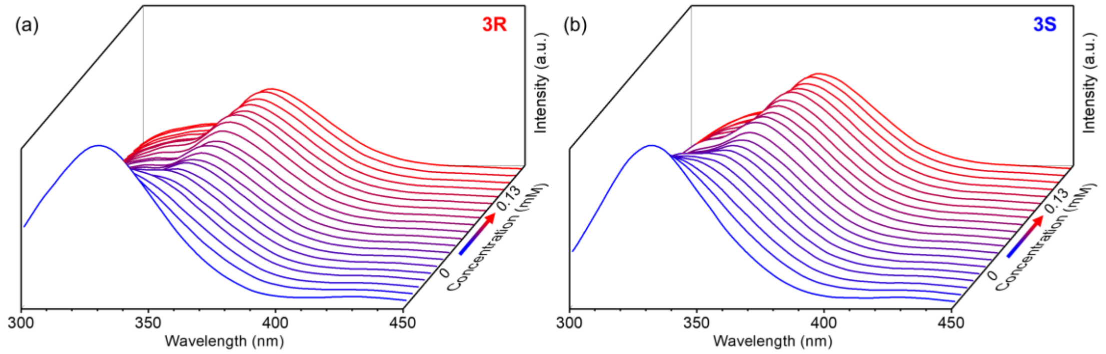

Figure S10. Luminescence spectra of PCN-700-C with the additions of 3R (a) and 3S (b).

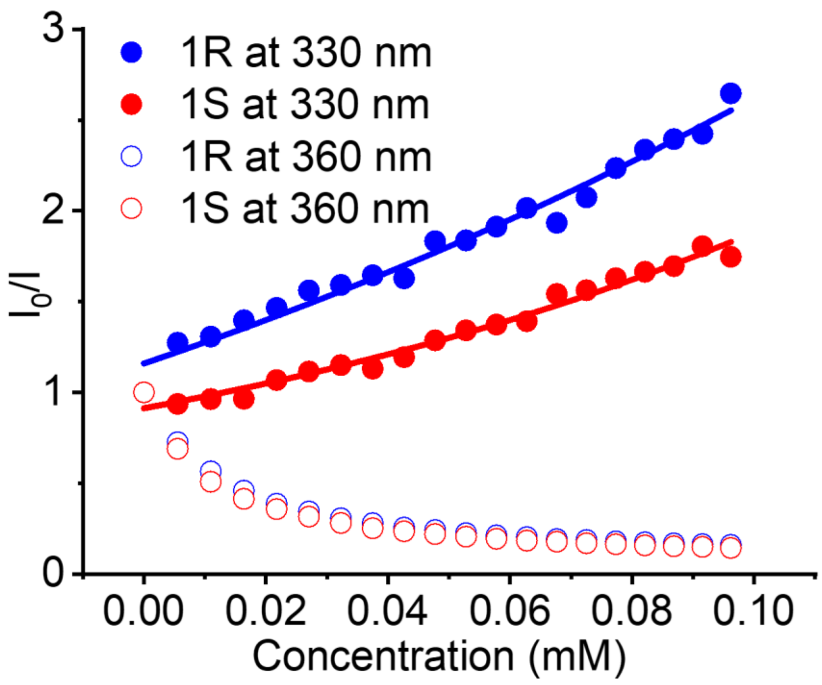

Figure S11. Luminescence intensity changes of PCN-700-C with the additions of 1R and 1S.

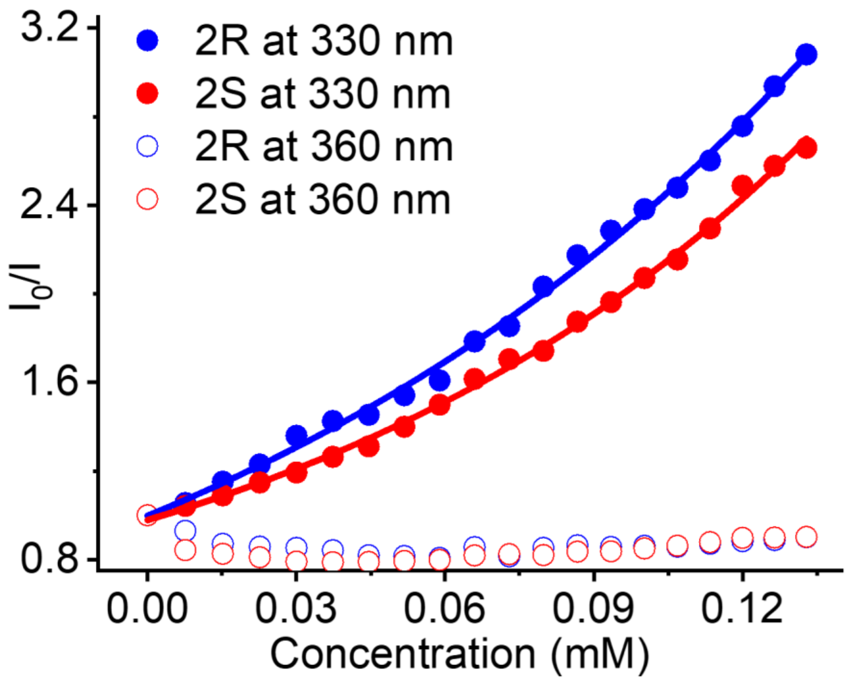

Figure S12. Luminescence intensity changes of PCN-700-C with the additions of 2R and 2S.

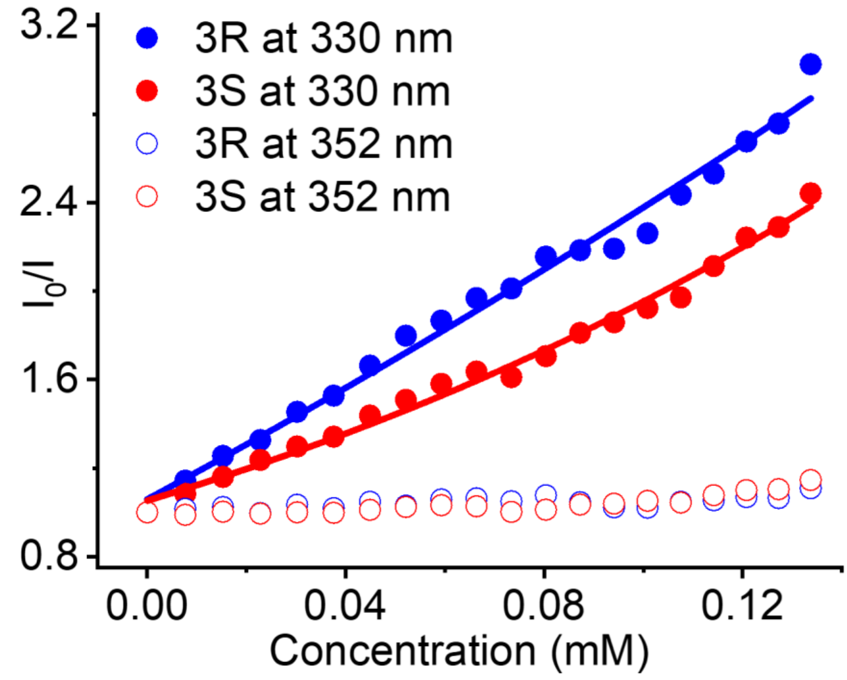

Figure S13. Luminescence intensity changes of PCN-700-C with the additions of 3R and 3S.

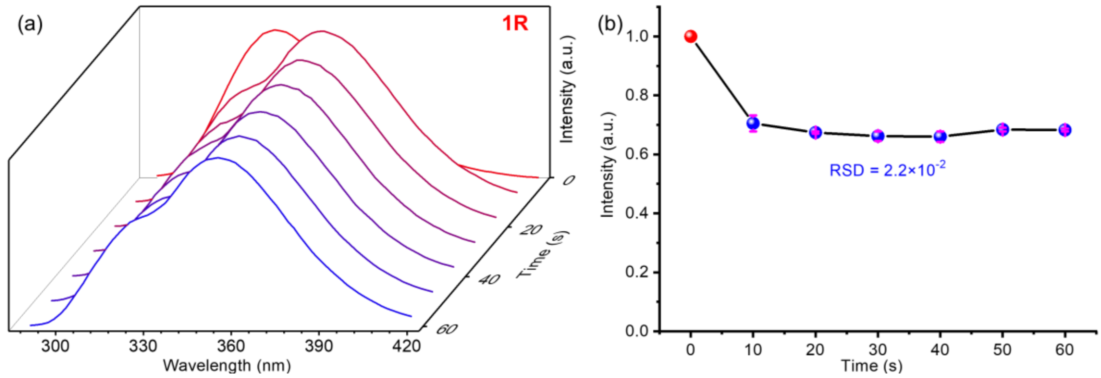

Figure S14. Time-dependant luminescence spectra (a) and intensity changes (b) of PCN-700-C with the additions of 1R.

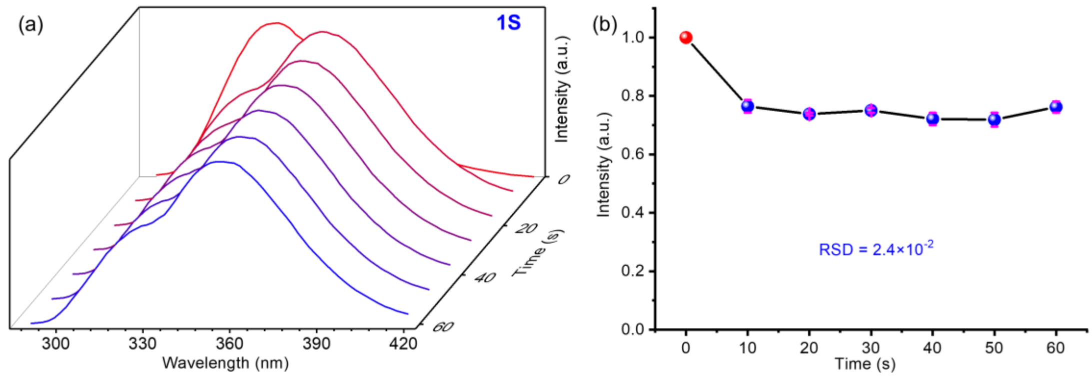

Figure S15. Time-dependant luminescence spectra (a) and intensity changes (b) of PCN-700-C with the additions of 1S.

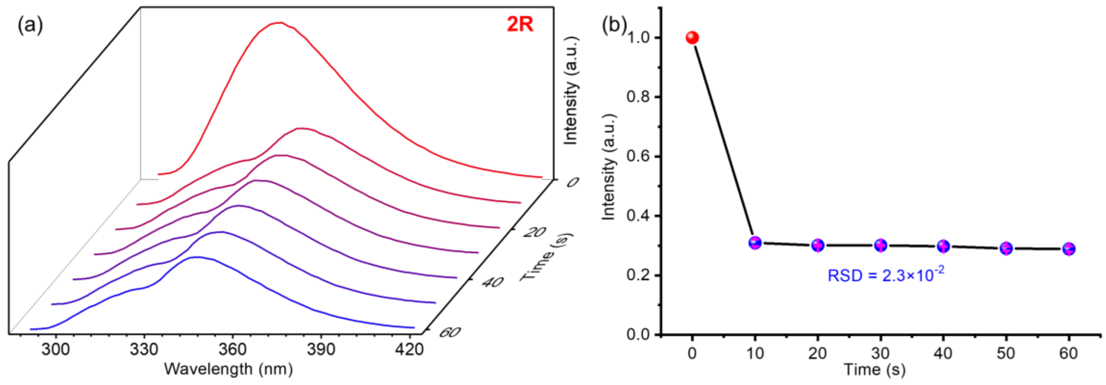

Figure S16. Time-dependant luminescence spectra (a) and intensity changes (b) of PCN-700-C with the additions of 2R.

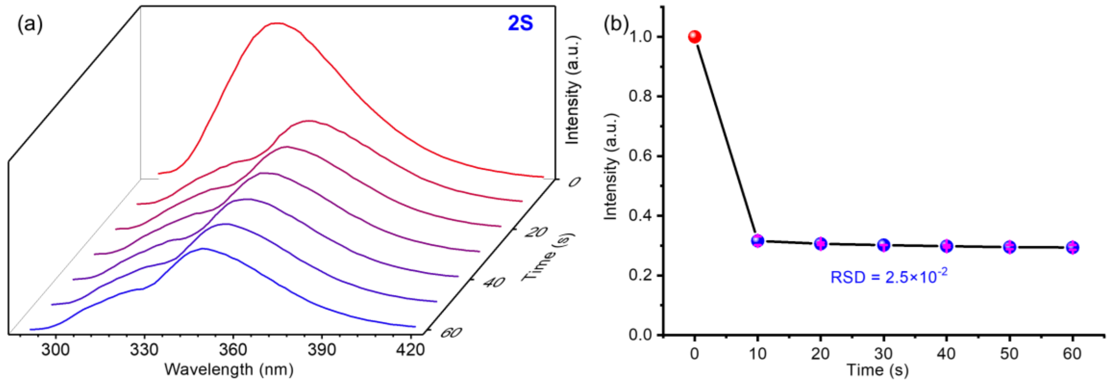

Figure S17. Time-dependant luminescence spectra (a) and intensity changes (b) of PCN-700-C with the additions of 2S.

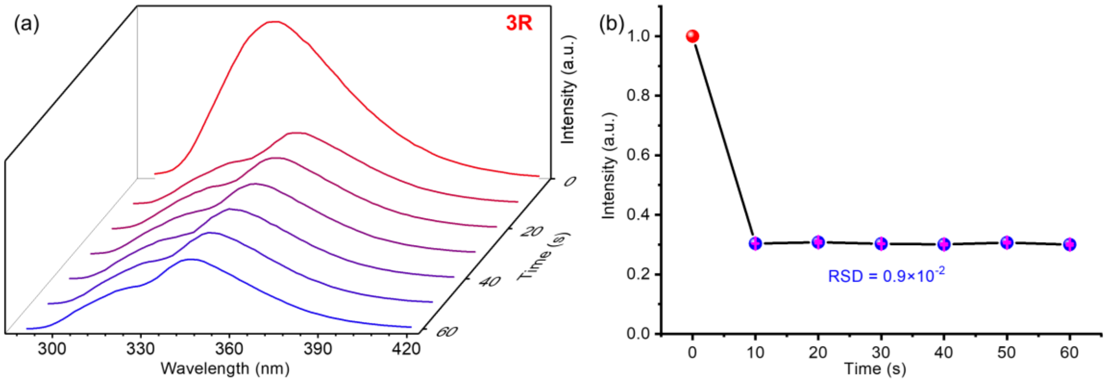

Figure S18. Time-dependant luminescence spectra (a) and intensity changes (b) of PCN-700-C with the additions of 3R.

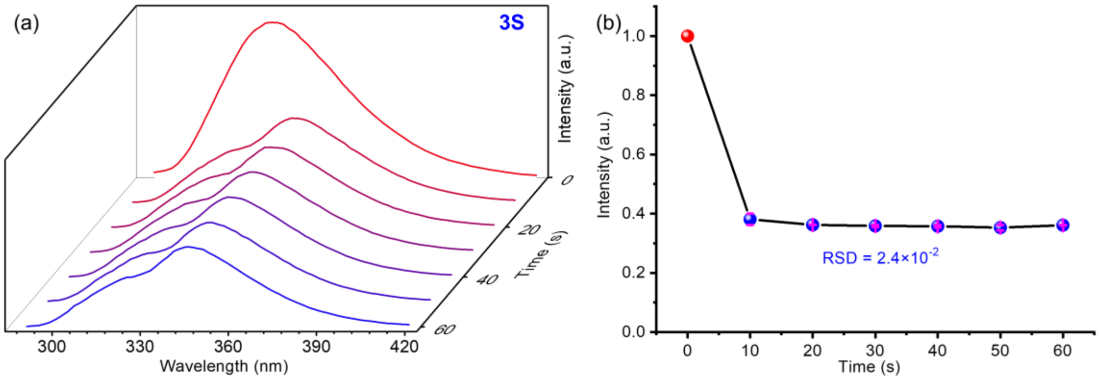

Figure S19. Time-dependant luminescence spectra (a) and intensity changes (b) of PCN-700-C with the additions of 3S.

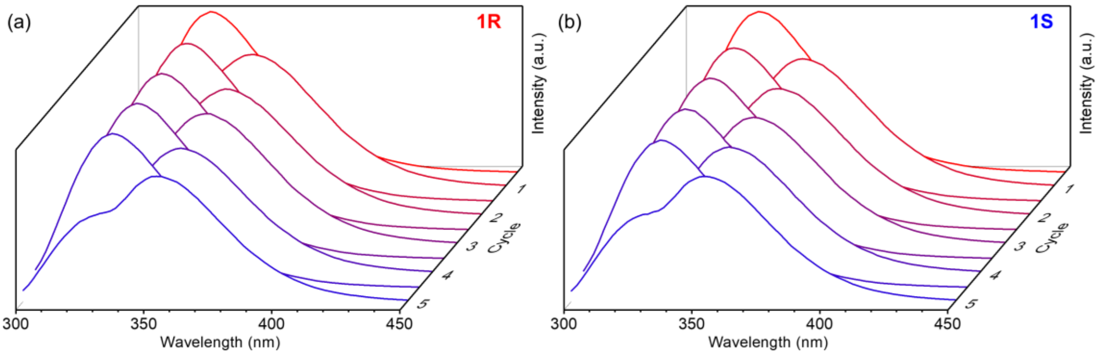

Figure S20. Luminescence spectra of the recycling tests of 1R (a) and 1S (b).

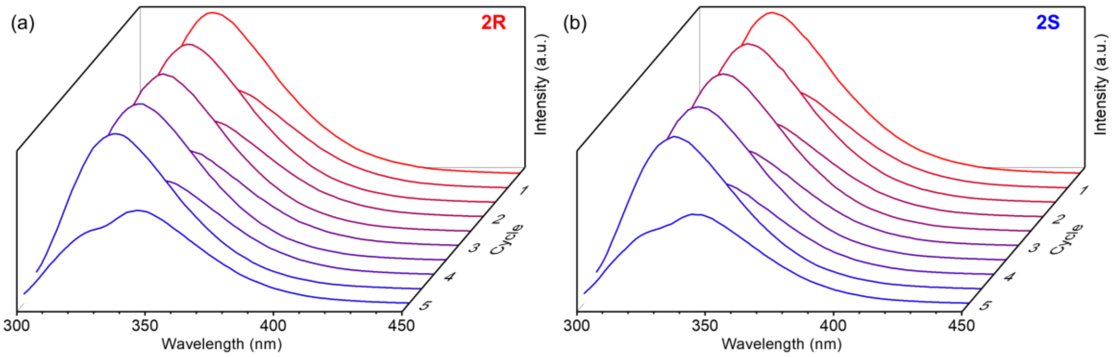

Figure S21. Luminescence spectra of the recycling tests of 2R (a) and 2S (b).

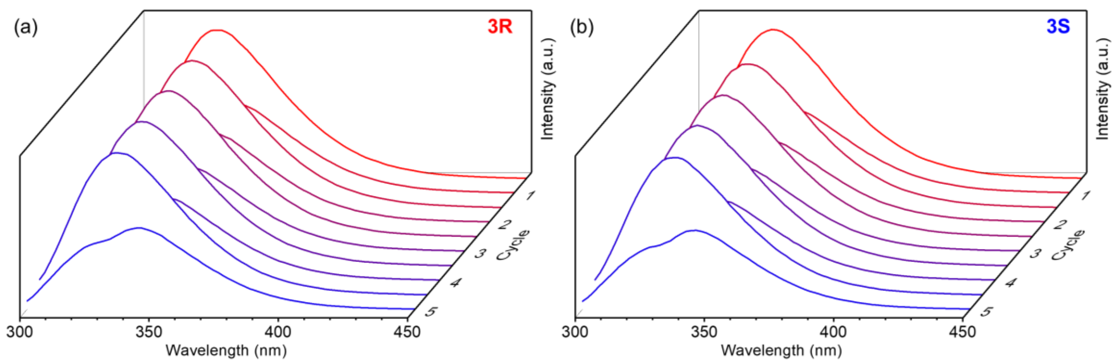

Figure S22. Luminescence spectra of the recycling tests of 3R (a) and 3S (b).

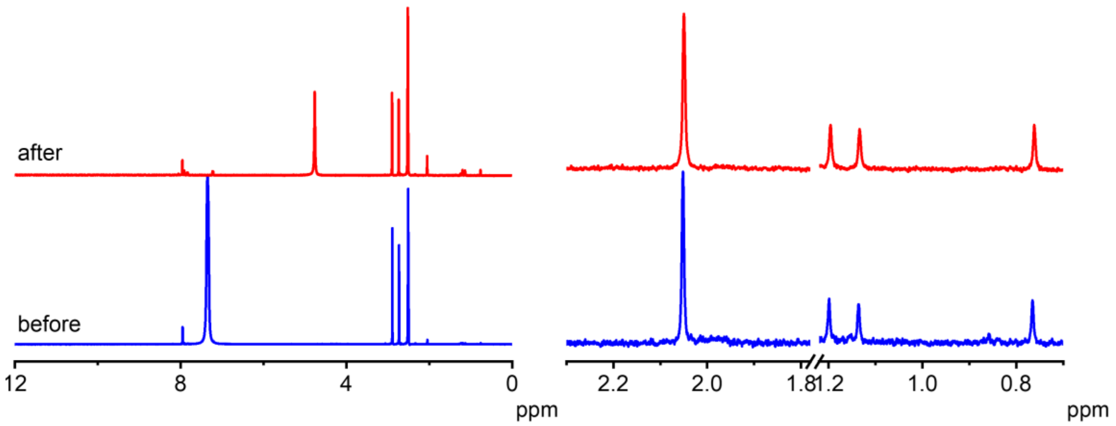

Figure S23. ^{1}H NMR spectra of PCN-700-C before and after the sensing experiments.

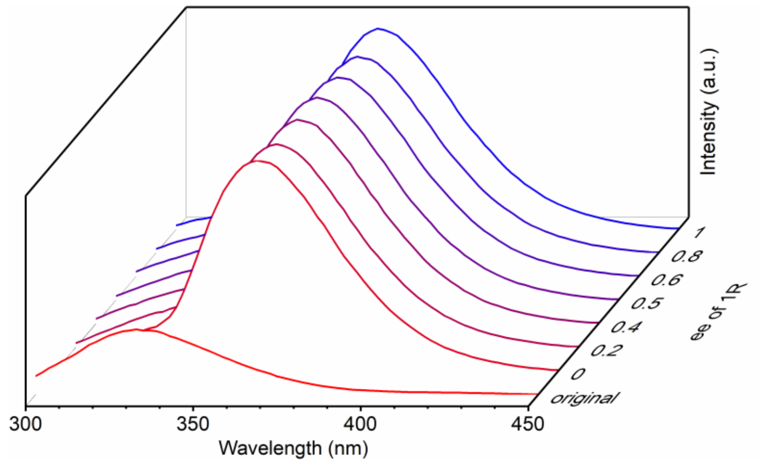

Figure S24. Emission spectra of PCN-700-C with the additions of different ee values of 1R.

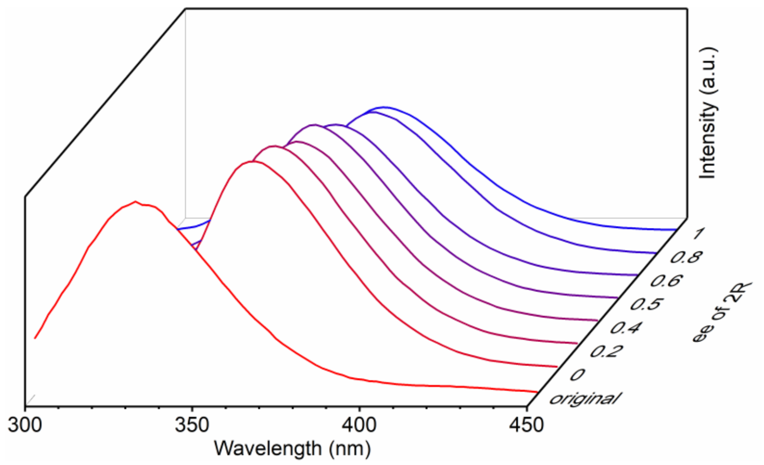

Figure S25. Emission spectra of PCN-700-C with the additions of different ee values of 2R.

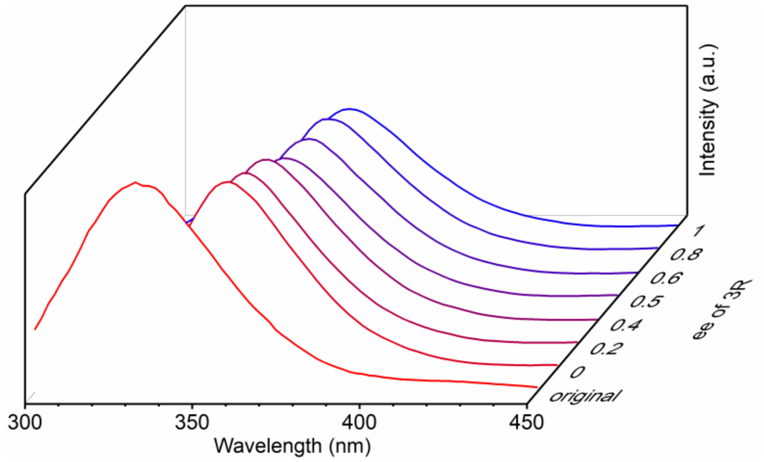

Figure S26. Emission spectra of PCN-700-C with the additions of different ee values of 3R.

Sensing Mechanism

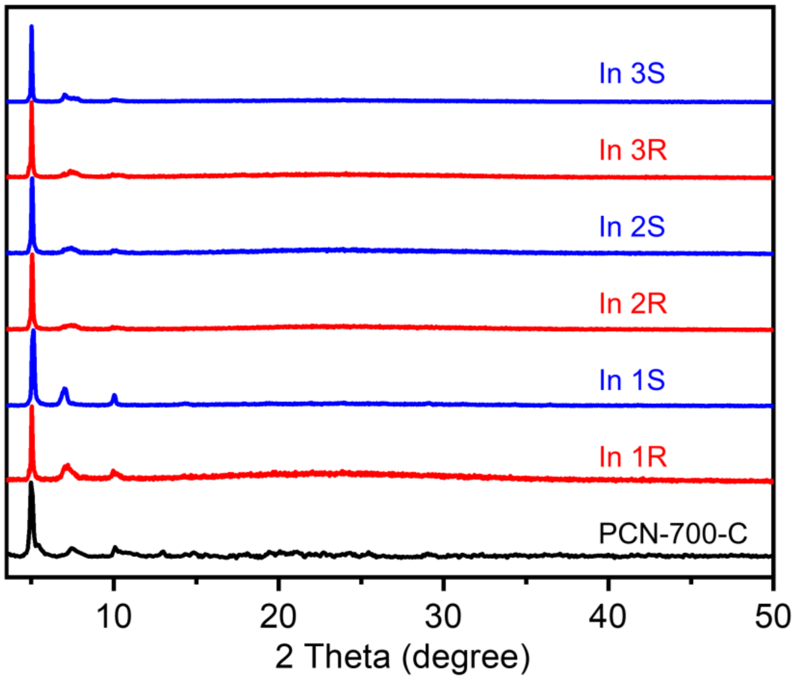

Figure S27. PXRD patterns of PCN-700-C after the sensing experiments.

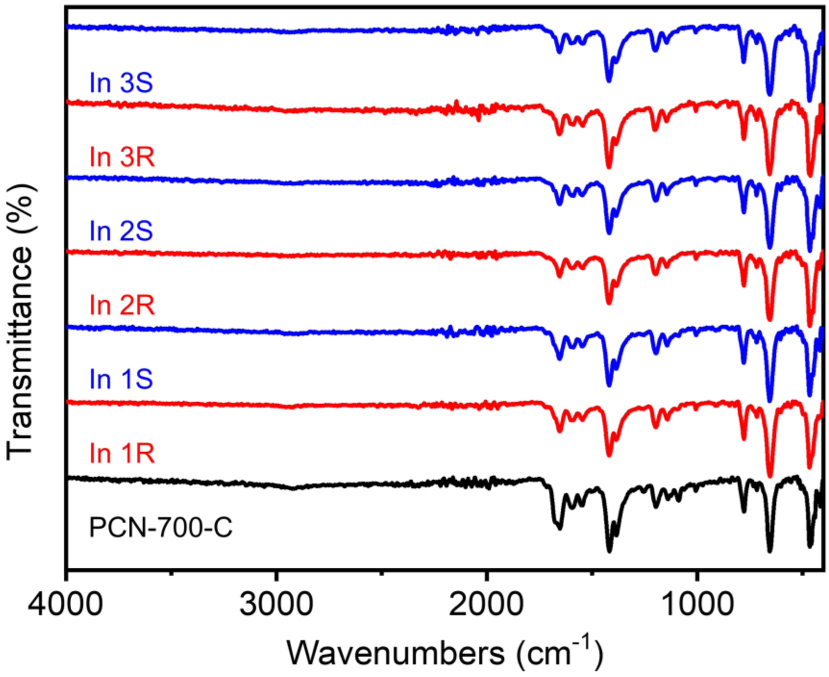

Figure S28. IR spectra of PCN-700-C after the sensing experiments.

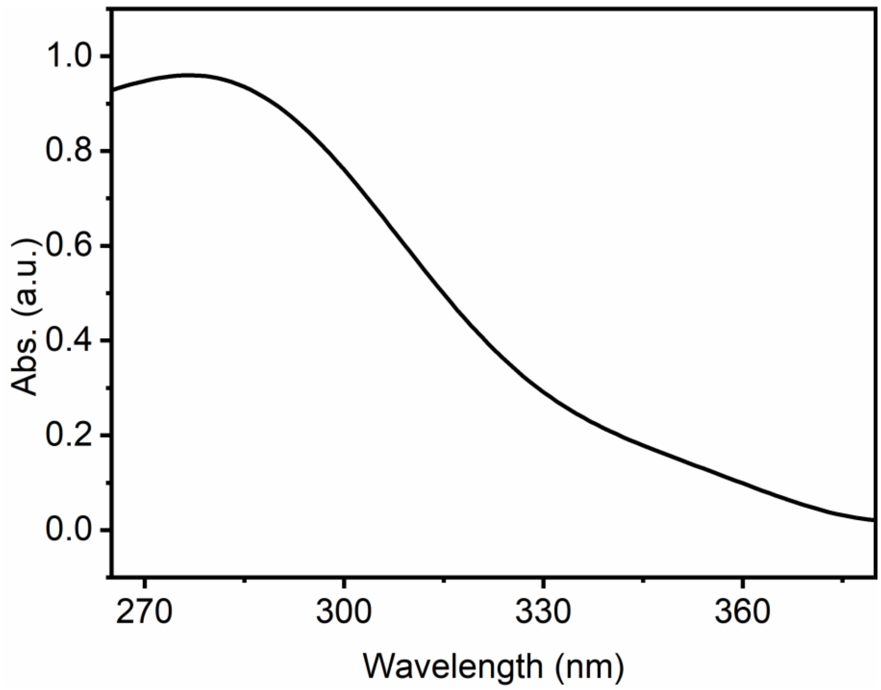

Figure S29. Liquid UV–vis absorption spectrum of the ligand.

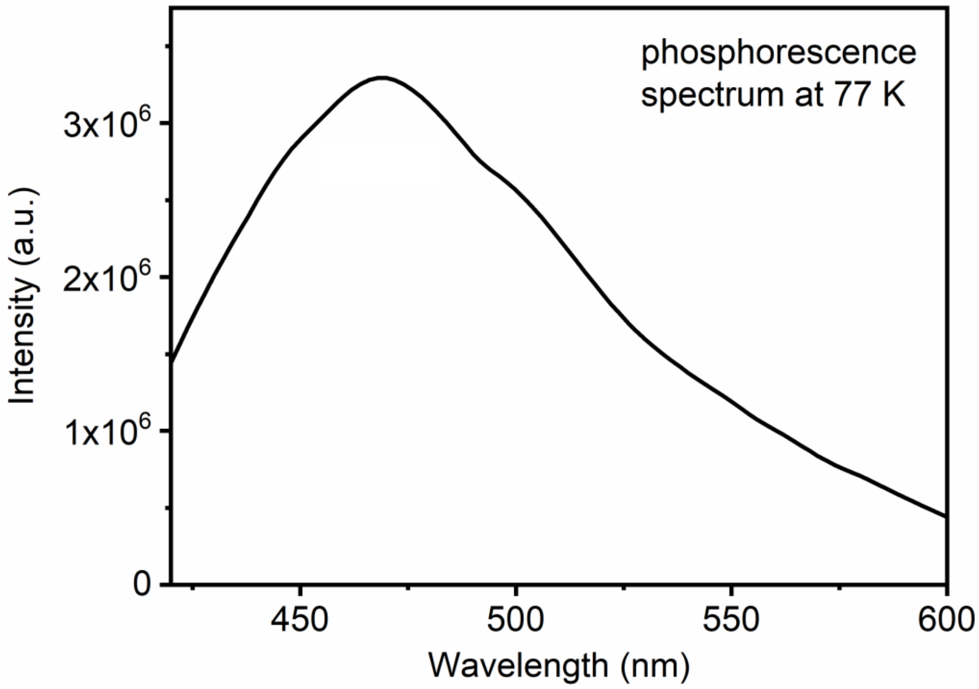

Figure S30. 77 K phosphorescence spectrum of PCN-700-C.

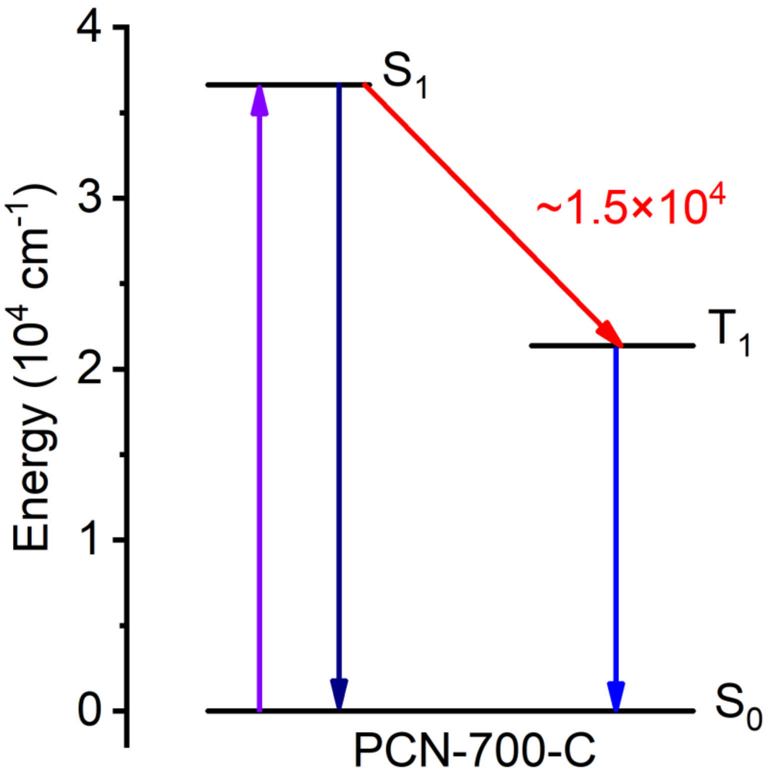

Figure S31. Luminescence mechanisum of PCN-700-C.

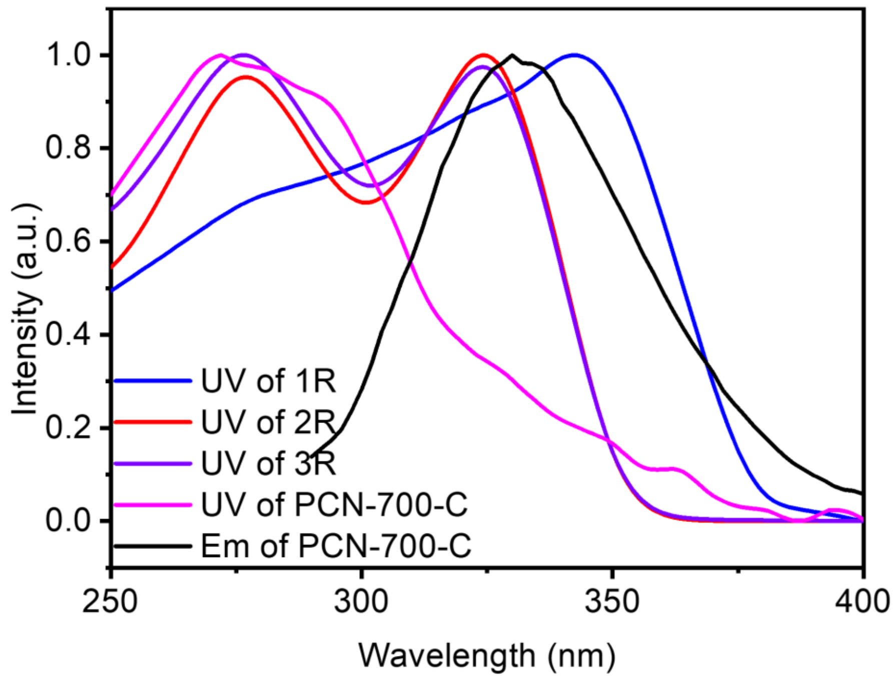

Figure S32. Liquid UV-vis spectra of the analytes and PCN-700-C, and the emission spectrum of PCN-700-C.

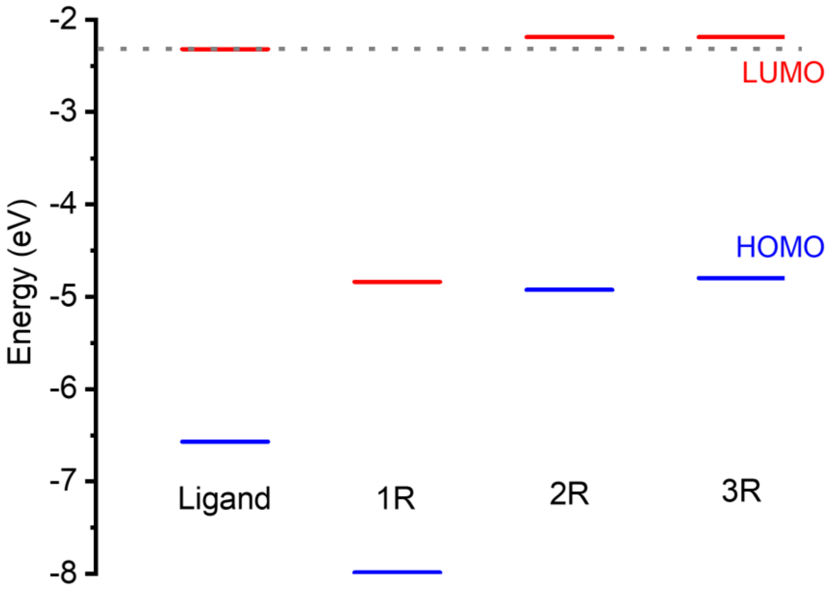

Figure S33. Energy levels of the ligand and the analytes.

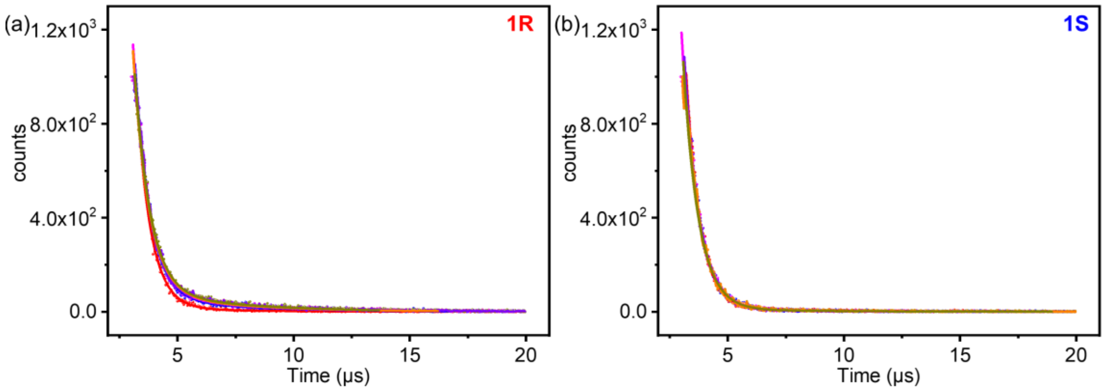

Figure S34. Lifetime spectra of PCN-700-C with the additions of 1R (a) and 1S (b).

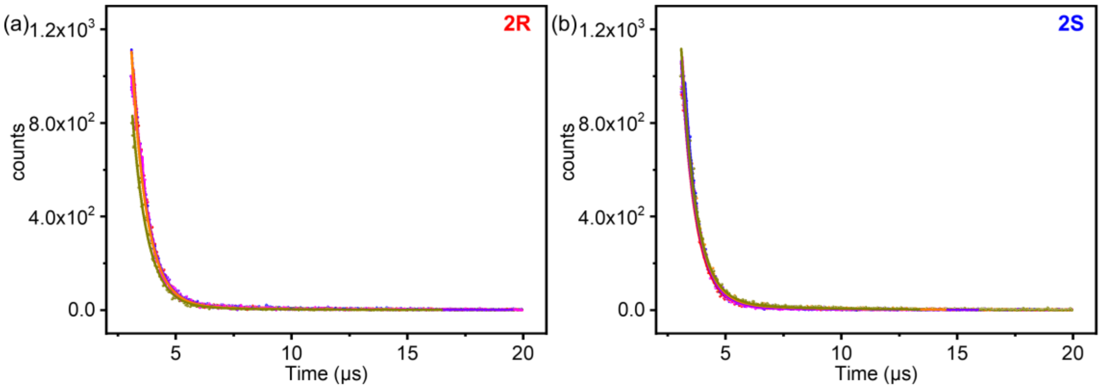

Figure S35. Lifetime spectra of PCN-700-C with the additions of 2R (a) and 2S (b).

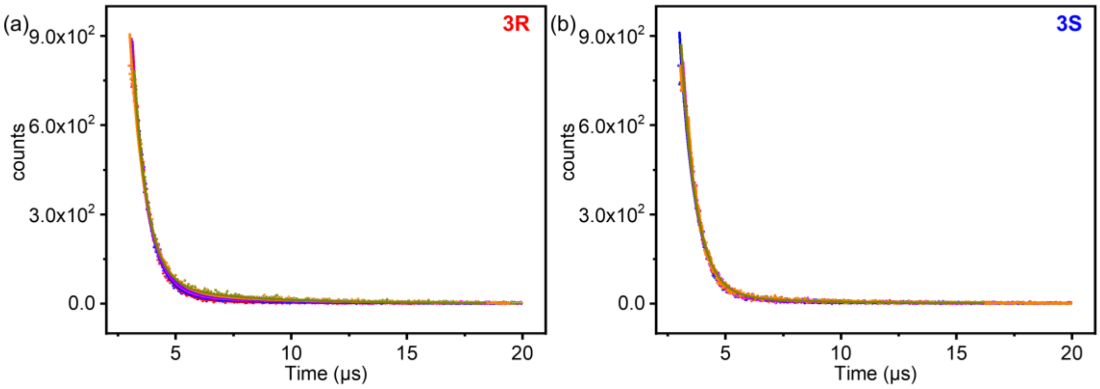

Figure S36. Lifetime spectra of PCN-700-C with the additions of 3R (a) and 3S (b).

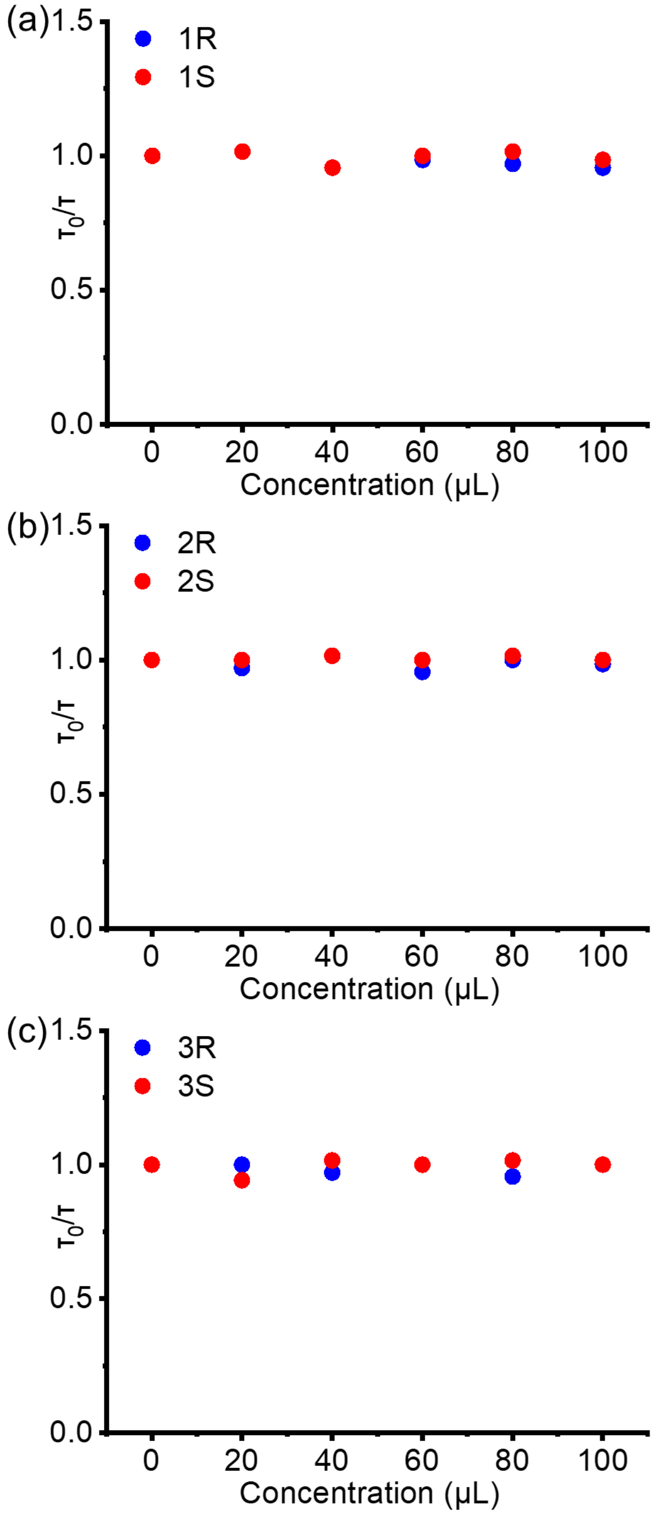

Figure S37. Lifetime changes of PCN-700-C with the additions of 1R/1S (a), 2R/2S (b), and 3R/3S (c). RSD values for 1R 2.2×10^{-2}; 1S 2.0×10^{-2}; 2R 2.0×10^{-2}; 2S 7.3×10^{-3}; 3R 1.8×10^{-2}; 3S 2.4×10^{-2}.

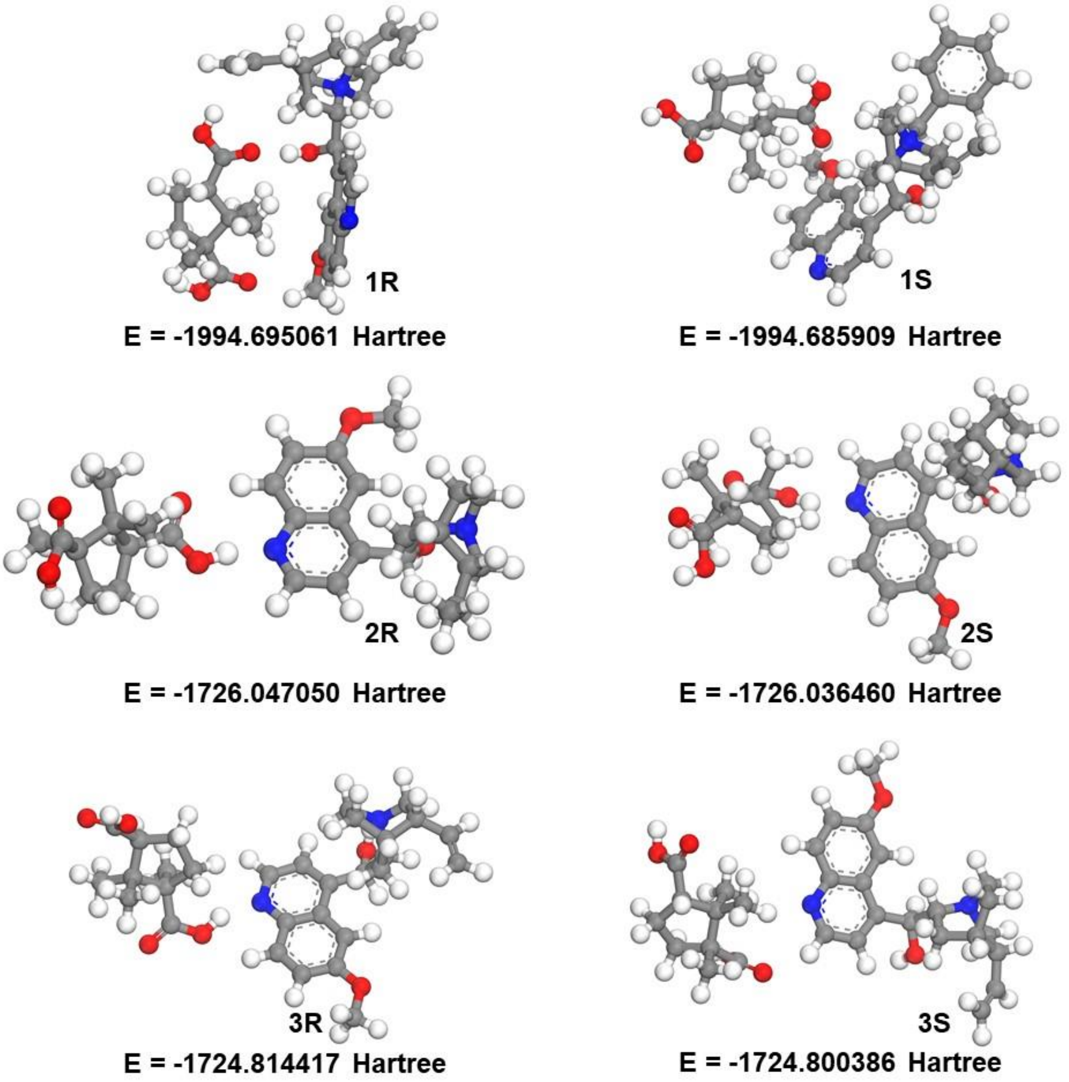

Figure S38. Single point energy calculation of the combination of D-camphorate linker and chiral substrates, namely 1R/1S, 2R/2S, and 3R/3S. The calculations with various forms of DFT were done with the TURBOMOLE suite.[4] The combinations were optimized by using the PBE0 density functional[5] together with the def-SV(P) basis set.[6] All geometry optimizations were converged. The vibration frequency calculations verified the stationary points were true minima.

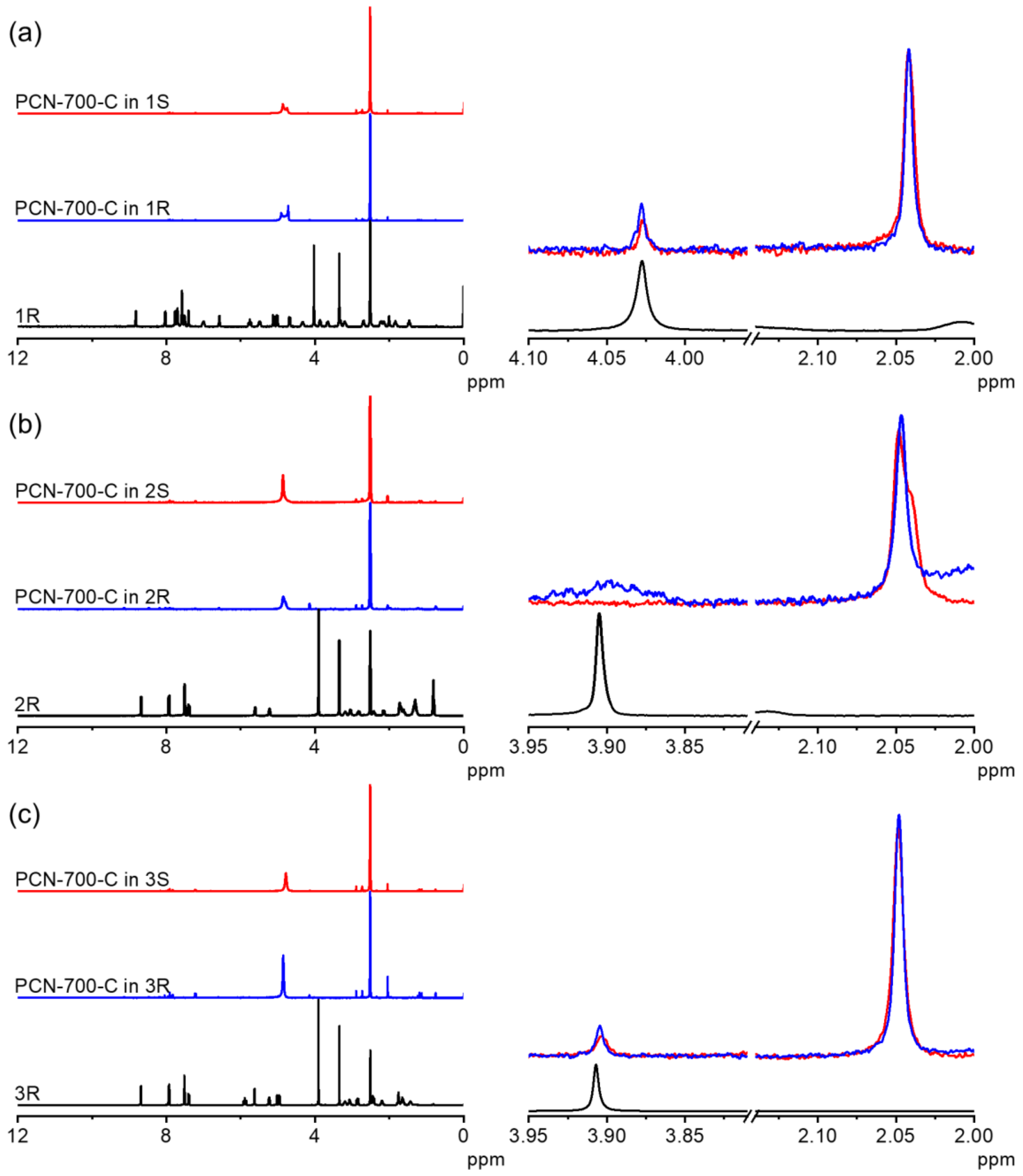

Figure S39. ^{1}H NMR spectra of PCN-700-C after soaking in 1R/1S (a), 2R/2S (b), and 3R/3S (c).

Tables

Table S1. Crystallographic data and structure refinement details.

	**PCN-700**	**PCN-700-C**
CCDC	2311789	2320626
Formula	$C_{32}H_{24}O_{16}Zr_3$	$C_{37}H_8O_{16}Zr_3$
Formula wt	938.17	982.09
Temperature (K)	110.00(10)	110.00(10)
Crystal System	tetragonal	tetragonal
Space Group	$P4_2/mmc$	$P4_2/mmc$
a (Å)	24.3510(15)	24.5205(9)
b (Å)	24.3510(15)	24.5205(9)
c (Å)	14.681(3)	13.9247(13)
α (deg)	90	90
β (deg)	90	90
γ (deg)	90	90
Z	4	4
V (Å^3)	8705.5(18)	8372.3(10)
ρ_{calc} (g cm^{-3})	0.716	0.779
μ (mm^{-1})	0.382	3.301
F (000)	1856.0	1912.0
R_{int}	0.1523	0.1252
$^aR_1$, $^bwR_2$ ($I > 2\sigma(I)$)	0.0974, 0.2547	0.0793, 0.2282
$^aR_1$, $^bwR_2$ (all data)	0.1391, 0.2923	0.1098, 0.2523

$^aR_1 = \Sigma||F_o| - |F_c||/\Sigma|F_o|$, $^bwR_2 = [\Sigma w(F_o^2 - F_c^2)^2/\Sigma w(F_o^2)^2]^{1/2}$

Table S2. Selected enantioselective luminescence sensors.

Materials	Type	Analytes	K_1 / M^{-1}	K_2 / M^{-1}	K_1/K_2	Re.
PCN-700-C	MOF	*N*-benzylquininium chloride	1.23×10^5	8.31×10^4	1.48	this work
		hydroquinine	1.77×10^4	1.22×10^4	1.45	
		quinine	1.22×10^4	8.11×10^3	1.50	
dendrimer	molecule	phenylalaninol	5.6×10^2	4.7×10^2	1.18	7
BINOL-2	molecule	2-amino-3-methyl -1-butanol	4.7×10	4.3×10	1.09	8
BINOL-3	molecule	2-amino-3-methyl -1-butanol	6.3×10	5.7×10	1.11	
BINOL-4	molecule	2-amino-3-methyl -1-butanol	8.3×10	8.0×10	1.04	
BINOL-2	molecule	2-amino-4-methyl -1-pentanol	6.1×10	5.4×10	1.13	
BINOL-3	molecule	2-amino-4-methyl -1-pentanol	9.2×10	7.3×10	1.26	
BINOL-4	molecule	2-amino-4-methyl -1-pentanol	9.0×10	8.2×10	1.10	
BINOL-2	molecule	2-amino-3-phenyl -1-propanol	3.1×10^2	2.5×10^2	1.21	
BINOL-3	molecule	2-amino-3-phenyl -1-propanol	4.5×10^2	3.5×10^2	1.27	
BINOL-4	molecule	2-amino-3-phenyl -1-propanol	5.2×10^2	4.3×10^2	1.22	
BINOL-4	molecule	2-amino-3-phenyl -1-propanol	5.6×10^2	4.6×10^2	1.22	
bisbinaphthyl-based molecule	molecule	mandelic acid	3.5×10^2	1.6×10^2	2.13	9
bisbinaphthyl-based molecule	molecule	mandelic acid	-	-	-	10
propanolol amide calix[4]arene	molecule	phenylalaninol	1.7×10^{-1}	9.0×10^{-2}	1.90	11
$[Zn_8(L_1)_4Cl_8]\cdot5THF$	cage	alanine	1.2×10^4	3.4×10^3	3.69	12
$\{[Cd_2(L_2)(H_2O)_2]\cdot6.5DMF\cdot3EtOH\}_n$	MOF	2-amino-1-propanol	1.9×10^4	1.6×10^4	1.25	13
		2-amino-2-phenylethanol	3.1×10^4	2.7×10^4	1.17	
		2-amino-3-phenylpropanol	6.8×10^2	4.9×10^2	1.39	
		2-amino-3-methyl -1-butanol	1.66×10^3	5.3×10^2	3.12	
$[Zn_8(L_3)_4I_8]\cdot4MeOH\cdot4H_2O$	cage	1-phenylethylamine	-	-	2.87	14
		1-phenylpropylamine	-	-	1.32	
		1-(4-methylphenyl) ethylamine	-	-	1.30	
		1-(4-chlorophenyl) ethylamine	-	-	1.89	
Eu octadentate cyclen (tetraaza-12-crown-4)	complex	*N*-Boc-aspartate	-	-	1.33	15
binaphthalene boron-dipyrromethene conjugate	molecule	1-phenylethylamine	2.3×10^2	1.6×10^2	1.40	16
CdTe QDs	QD	cysteine	-	-	-	17
binaphthol-based crown ether derivatives	molecule	secondary ammonium ion	-	-	-	18

CCOF 7	COF	α-pinene	1.3×10^3	4.0×10^2	3.49	19
Zn-MOF-C-Tb	MOF	Cinchonine	4.7×10^3	3.5×10^3	1.35	20
		N-benzylcinch onium chloride	4.5×10^3	3.4×10^3	1.33	
		2-amino-1-butanol	5.6×10^3	2.6×10^3	2.16	
		2-amino-1-propanol	7.2×10	4.7×10	1.53	
Cd-MOF-C-Eu	MOF	quinine	5.9×10^3	4.9×10^3	1.20	21
D-AP@UiO-66-(COOH)$_2$	MOF	phenylalaninol	1.6×10^3	8.4×10^2	1.89	22
L-AP@UiO-66-(COOH)$_2$	MOF	phenylalaninol	1.5×10^2	1.0×10^2	1.50	
NKU-1000-C-Tb	hydrogen-bonded framework	2-amino-1-propanol	7.3×10^2	6.4×10^2	1.14	23
		1,2-propanediol	9.1×10^5	5.9×10^5	1.54	

H_2L_1: 6,6'-((((1*R*,2*R*)-cyclohexane-1,2-diyl)bis(azanediyl))bis(methylene))bis(4-(*tert*-butyl)-2-((*E*)-2-(pyridin-4-yl)vinyl)phenol); H_4L_2: 4,4',4'',4'''-(2,2'-dihydroxy-[1,1'-binaphthalene]-4,4',6,6'-tetrayl)tetrabenzoic acid; H_2L_3: 4-(*tert*-butyl)-2-((*E*)-(((1*R*,2*R*)-2-((5-(*tert*-butyl)-2-hydroxy-3-((*E*)-2-(pyridin-4-yl)vinyl)benzyl)amino)cyclohexyl)imino)methyl)-6-((*E*)-2-(pyridin-4-yl)vinyl)phenol; AP: amino propanol; COF: covalent organic framework; QD: quantum dot.

References

(1) Sheldrick, G. M. Crystal structure refinement with SHELXL. *Acta Crystallogr., Sect. C: Struct. Chem.* **2015**, *71*, 3-8.

(2) Sheldrick, G. M. A short history of SHELX. *Acta Crystallogr., Sect. A: Found. Crystallogr.* **2008**, *64*, 112-122.

(3) Yuan, S.; Chen, Y.-P.; Qin, J.-S., Lu, W.; Zou, L.; Zhang, Q.; Wang, X.; Sun, X.; Zhou, H.-C. Linker installation: engineering pore environment with precisely placed functionalities in zirconium MOFs. *J. Am. Chem. Soc.* **2016**, *138*, 8912-8919.

(4) Ahlrichs, R. et al. TURBOMOLE 6.3.1, University of Karlsruhe, **2012**, http://www.turbomole.com, accessed May 19, 2016

(5) Adamo, C.; Barone, V. Toward reliable density functional methods without adjustable parameters: The PBE0 model. *J. Chem. Phys.* **1999**, *110*, 6158-6170.

(6) Schäfer, A.; Horn, H.; Ahlrichs, R. Fully optimized contracted gaussian basis sets for atoms Li to Kr. *J. Chem. Phys.* **1992**, *97*, 2571-2577.

(7) Pugh, V. J.; Hu, Q. S.; Pu, L. The first dendrimer-based enantioselective fluorescent sensor for the recognition of chiral amino alcohols. *Angew. Chem. Int. Ed.* **2000**, *39*, 3638-3641.

(8) Pugh, V. J.; Hu, Q. S.; Zuo, X.; Lewis, F. D.; Pu, L. Optically active BINOL core-based phenyleneethynylene dendrimers for the enantioselective fluorescent recognition of amino alcohols. *J. Org. Chem*. **2001**, *66*, 6136-6140.

(9) Lin, J.; Hu, Q. S.; Xu, M. H.; Pu, L. A practical enantioselective fluorescent sensor for mandelic acid. *J. Am. Chem. Soc.* **2002**, *124*, 2088-2089.

(10) Xu, M. H.; Lin, J.; Hu, Q. S.; Pu, L. Fluorescent sensors for the enantioselective recognition of mandelic acid: signal amplification by dendritic branching. *J. Am. Chem. Soc.* **2002**, *124*, 14239-14246.

(11) Lynama, C.; Diamond, D. Varying solvent polarity to tune the enantioselective quenching of a calixarene host. *J. Mater. Chem*. **2005**, *15*, 307-314.

(12) Xuan, W.; Zhang, M.; Liu, Y.; Chen, Z.; Cui, Y. A chiral quadruple-stranded helicate cage for enantioselective recognition and separation. *J. Am. Chem. Soc.* **2012**, *134*, 6904-6907.

(13) Wanderley, M. M.; Wang, C.; Wu, C. D.; Lin, W. B. A chiral porous metal-organic framework for highly sensitive and enantioselective fluorescence sensing of amino alcohols. *J. Am. Chem. Soc*. **2012**, *134*, 9050-9053.

(14) Dong, J.; Zhou, Y.; Zhang, F.; Cui, Y. A highly fluorescent metallosalalen-based chiral cage for enantioselective recognition and sensing. *Chem. -Eur. J.* **2014**, *20*, 6455-6461.

(15) Ito, H.; Shinoda, S. Chirality sensing and size recognition of n-boc-amino acids by cage-type dimeric lanthanide complexes: chirality detection of N-Boc-aspartate anions via luminescence colour change. *Chem. Commun.* **2015**, *51*, 3808-3811.

(16) Pujari, C.; Arindam, M.; Govardhan, S.; Jarugu, N. M. Remarkably selective and enantiodifferentiating sensing of histidine by a fluorescent homochiral Zn-MOF based on pyrene-tetralactic acid. *Chem. Sci.* **2016**, *7*, 3085-3091.

(17) Ghasemi, F.; Hormozi-Nezhad, M. R.; Mahmoudi, M. Time-resolved visual chiral discrimination of cysteine using unmodified Cdte quantum dots. *Sci. Rep.* **2017**, *7*, 890.

(18) Gangopadhyay, M.; Maity, A.; Dey, A.; Rajamohanan, P. R.; Ravindranathan, S.; Das, A. Chiral discrimination through ^{1}H NMR and luminescence spectroscopy: dynamic processes and solid strip for chiral recognition. *Chem. -Eur. J.* **2017**, *23*, 18303-18313.

(19) Wu, X.; Han, X.; Xu, Q.; Liu, Y.; Yuan, C.; Yang, S.; Liu, Y.; Jiang, J.; Cui, Y. Chiral BINOL-based covalent organic frameworks for enantioselective sensing. *J. Am. Chem. Soc.* **2019**, *141*, 7081-7089.

(20) Han, Z.; Wang, K.; Guo, Y.; Chen, W.; Zhang, J.; Zhang, X.; Siligardi, G.; Yang, S.; Zhou, Z.; Sun, P.; Shi, W.; Cheng P. Cation-induced chirality in a bifunctional metal-organic framework for quantitative enantioselective recognition. *Nat. Commun.* **2019**, *10*, 5117.

(21) Liu, T.Y.; Qua, X.L.; Yan, B. A sensitive metal-organic framework nanosensor with cation-introduced chirality for enantioselective recognition and determination of quinine and quinidine in human urine. *J. Mater. Chem. C* **2020**, *8*, 14579-14586.

(22) Xiao, J.; Wang, X.; Xu, X.; Tian, F.; Liu, Z. Fabrication of a “turn-on”-type enantioselective fluorescence sensor via a modified achiral MOF: applications for synchronous detection of phenylalaninol enantiomers. *Analyst* **2021**, *146*, 937-942.

(23) Han, Z.; Wang, M.; Wang, K.; Cheng, P.; Shi, W. A bifunctional coordination-chain-based hydrogen-bonded framework for quantitative enantioselective sensing. *Chem. -Eur. J.* **2023**, *29*, e202301892.
